# The General Amino Acid Permease FfGap1 of *Fusarium fujikuroi* Is Sorted to the Vacuole in a Nitrogen-Dependent, but Npr1 Kinase-Independent Manner

**DOI:** 10.1371/journal.pone.0125487

**Published:** 2015-04-24

**Authors:** Andreas Pfannmüller, Dominik Wagner, Christian Sieber, Birgit Schönig, Mélanie Boeckstaens, Anna Maria Marini, Bettina Tudzynski

**Affiliations:** 1 Institut für Biologie und Biotechnologie der Pflanzen, Westfälische Wilhelms-Universität Münster, Münster, Germany; 2 Laboratoire de Biologie du Transport Membranaire, Institut de Biologie et de Médecine Moléculaires, Université Libre de Bruxelles, Gosselies, Belgium; 3 Institute of Bioinformatics and Systems Biology, Helmholtz Zentrum München, German Research Center for Environmental Health (GmbH), Neuherberg, Germany; Seoul National University, KOREA, REPUBLIC OF

## Abstract

The rice pathogenic fungus *Fusarium fujikuroi *is well known for the production of a broad spectrum of secondary metabolites (SMs) such as gibberellic acids (GAs), mycotoxins and pigments. The biosynthesis of most of these SMs strictly depends on nitrogen availability and of the activity of permeases of nitrogen sources, e.g. the ammonium and amino acid permeases. One of the three ammonium permeases, MepB, was recently shown to act not only as a transporter but also as a nitrogen sensor affecting the production of nitrogen-repressed SMs. Here we describe the identification of a general amino acid permease, FfGap1, among the 99 putative amino acid permeases (AAPs) in the genome of *F*. *fujikuroi*. FfGap1 is able to fully restore growth of the yeast *gap1∆* mutant on several amino acids including citrulline and tryptophane. In *S*. *cerevisiae*, Gap1 activity is regulated by shuttling between the plasma membrane (nitrogen limiting conditions) and the vacuole (nitrogen sufficiency), which we also show for FfGap1. In yeast, the Npr1 serine/threonine kinase stabilizes the Gap1 position at the plasma membrane. Here, we identified and characterized three *NPR1*-homologous genes, encoding the putative protein kinases FfNpr1-1, FfNpr1-2 and FfNpr1-3 with significant similarity to yeast Npr1. Complementation of the yeast *npr1Δ *mutant with each of the three *F*. *fujikuroi NPR1 *homologues, resulted in partial restoration of ammonium, arginine and proline uptake by *FfNPR1-1* while none of the three kinases affect growth on different nitrogen sources and nitrogen-dependent sorting of FfGap1 in *F*. *fujikuroi*. However, exchange of the putative ubiquitin-target lysine 9 (K9A) and 15 (K15A) residues of FfGap1 resulted in extended localization to the plasma membrane and increased protein stability independently of nitrogen availability. These data suggest a similar regulation of FfGap1 by nitrogen-dependent ubiquitination, but differences regarding the role of *Fusarium *Npr1 homologues compared to yeast.

## Introduction

In the phytopathogenic ascomycete *Fusarium fujikuroi*, the biosynthesis of a broad spectrum of secondary metabolites (SMs), such as the plant hormones gibberellins (GA) and the red polyketide pigments bikaverin and fusarubins [1,2,3], is strictly repressed, while the biosynthesis of mycotoxins such as fusarin C, fusaric acid, and apicidin F is induced by high nitrogen concentrations [4,5,6,7]. Therefore, mechanisms of nitrogen sensing and nitrogen-mediated regulation of gene expression are in the focus of our research.

Uptake and eventually sensing of nitrogen by specific permeases play key roles in nitrogen metabolism [8,9]. Processes of nitrogen sensing and subsequent transduction of the signal to a nitrogen regulation network have been intensively studied in yeast. Beside the three ammonium permeases Mep1 to Mep3, yeast have about 20 amino acid permeases (AAPs) [10]. The expression of nitrogen permease-encoding genes such as *GAP1* and the *MEP* genes is negatively influenced by the availability of favored nitrogen sources like glutamine, while poor nitrogen sources such as urea or proline support their transcription in a Gln3 and Gat1-dependent manner [11–14]. Some of the AAPs have been proposed to play a role as nitrogen sensors, e.g. the non-transporting amino acid carrier homologue Ssy1 [15] and the general amino acid permease Gap1 [16]. Yeast Mep2, and its orthologue from *Candida albicans* for instance, are proposed to play a signaling role as they are required for the filamentation process occurring upon nitrogen limitation [17–21].

We have characterized all three ammonium permease-encoding genes in *F*. *fujikuroi*, *MEPA*, *MEPB*, and *MEPC*, regarding their participation in ammonium uptake and their potential involvement in signal transduction. The expression of all three genes is strictly regulated by the general nitrogen regulator AreA, the homologue of *Saccharomyces cerevisiae* GATA transcription factors Gln3 and Gat1 [13,14,22]. Severe growth defects of Δ*MEPB* mutants on low ammonium medium and deregulation of several nitrogen-repressed genes, such as the GA and bikaverin biosynthetic genes, suggested that MepB could function as an ammonium sensor in addition to its important role in ammonium transport in *F*. *fujikuroi*. However, the mechanism of ammonium sensing by MepB is not yet understood.

Plasma membrane permease activity can be modulated by transcriptional regulation, control of cell surface protein level and fine-tuning of the inherent transport activity via post-translational modification. In yeast, the serine/threonine protein kinase Npr1 (nitrogen permease reactivator) is a regulator of the activity of permeases of nitrogenous compounds, being required for optimal uptake of nitrogen sources in cells grown on a non-preferred nitrogen source [21,23,24]. Npr1 is thus required for the activity of several permeases including the general amino acid permease Gap1 [24,25]. In the latter case, Npr1 is required to maintain Gap1 at the cytoplasmic membrane by preventing ubiquitination-dependent endocytosis under non-preferred nitrogen supply. In the *npr1* mutant, newly synthesized Gap1 is further directly sorted to the vacuole and never reaches the plasma membrane whatever the quality of the nitrogen supply [26–28]. As a consequence, the uptake of citrulline, ornithine and tryptophan, all depending on the activity of Gap1, is affected in the *npr1* mutant. Beside the stabilization/destabilization of nitrogen permeases, there seem to be other ways for Npr1 to affect the activity of specific permeases. Npr1 is required for the inherent activity of ammonium transport by Mep1, Mep2 and Mep3, *the* npr1 mutant exhibiting a growth defect on low-ammonium medium similar to that of a mutant lacking the three ammonium permeases [18,25,29]. However, contrary to Gap1, plasma membrane localization of the ammonium permease Mep2 is not altered in the Δ*NPR1* mutant of *S*. *cerevisiae* and *Candida albicans* [20,29,30] Under poor nitrogen supply, the Npr1 kinase mediates phosphorylation of the serine residue S457 in the C-terminal auto-inhibitory domain of Mep2 [29]. This phosphorylation relieves auto-inhibition and allows Mep2 to adopt an active conformation that enables high ammonium transport capacity. Due to the impairment of uptake of repressing nitrogen sources, a number of genes sensitive to nitrogen catabolite repression (NCR) are indirectly derepressed in Npr1-lacking cells growing in the presence of sufficient ammonium or glutamine [25,31]. The level of Npr1 phosphorylation, and presumably of its activity, is controlled by TORC1 (target of rapamycin complex1) according to the quality of the nitrogen supply [30,32–34]. Treatment of yeast cells with the TORC1-inhibiting drug rapamycin as well as nitrogen starvation promote rapid though not complete dephosphorylation of Npr1, while the protein is hyperphosphorylated in a nitrogen-rich medium [34].

Not much is known about mechanisms for regulating subcellular localization of nitrogen permeases in filamentous fungi. So far no Npr1-like regulators have been characterized, and therefore, their putative involvement in regulating the activity of any nitrogen permease has not been studied in any filamentous fungus.

In this work, we identified a homolog of the yeast Gap1, FfGap1, among the 99 annotated putative amino acid permeases in the genome of *F*. *fujikuroi* by complementing the *S*. *cerevisiae gap1Δ* mutant. We show that *FfGAP1* is not expressed in media containing glutamine, and that its transcription strictly depends on the presence of the GATA transcription factor AreA. The FfGap1 protein is localized to the plasma membrane when internal amino acid levels are low. Upon addition of glutamine, the FfGap1-Gfp fusion protein is endocytosed and delivered to the vacuole. To test whether Npr1-like protein kinases are required for stabilization of FfGap1 at the plasma membrane as in yeast, we cloned three homologous genes of *ScNpr1*, *FfNPR1-1*, *FfNPR1-2* and *FfNPR 1–3*, and studied their role in regulating FfGap1 activity. We show that complementation of the yeast *npr1Δ* mutant with the *F*. *fujikuroi NPR1-1*, *NPR1-2* and *NPR1-3* genes, respectively, resulted in partial restoration of growth on ammonium, arginine and proline by *FfNPR1-1*. However, the putative serine/threonine kinases had no impact on sorting of FfGap1. Instead, we demonstrate that two conserved lysine residues at the N-terminus of FfGap1 are involved in regulating FfGap1 protein abundance and its sorting to the vacuoles. Point mutations of these lysine residues, K7 and K15, of FfGap1 resulted in prolonged maintenance at the cytoplasmic membrane under high glutamine conditions, probably by preventing ubiquitination-dependent endocytosis.

## Results

### Identification of a *F*. *fujikuroi* Gap1 homologue

In order to identify a potential orthologue of the *S*. *cerevisiae* Gap1, we retrieved 99 predicted amino acid permeases (AAPs) of the recently sequenced genome of *F*. *fujikuroi* IMI 58289 [7] (S1 Table). A BLAST [35] search identified 19 of the 99 *F*. *fujikuroi* AAPs with significant similarity (E-value < 1e-50, identity > 25%) to at least one of four known general amino acid permeases (GAPs) of *S*. *cerevisiae* [36], *Candida albicans* [37], *Neurospora crassa* [38] and *Penicillium chrysogenum* [39].

**Fig 1:**
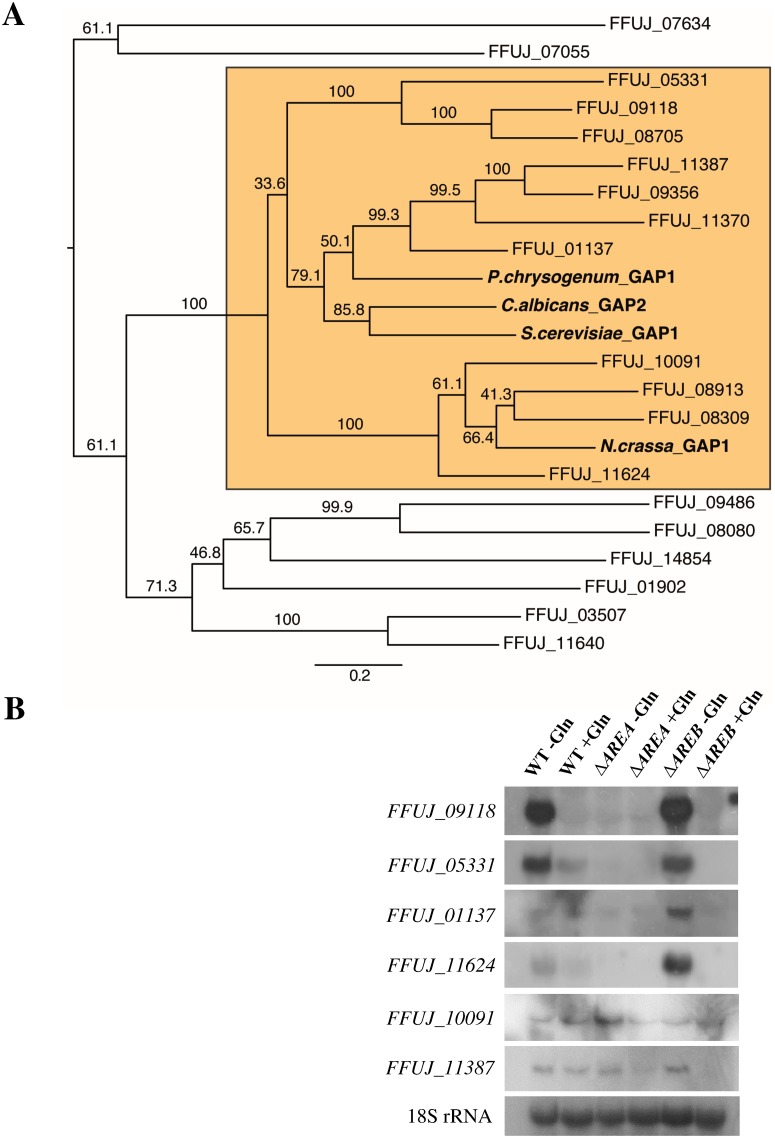
Identification of potential general amino acid permeases in *F*. *fujikuroi*. (A) Maximum likelihood tree showing the phylogenetical relationship between the general amino acid permeases (GAP) from *S*. *cerevisiae* [36], *C*. *albicans* [37], *N*. *crassa* [38], *P*. *chrysogenum* [39], and 19 *F*. *fujikuroi* proteins with the highest sequence homology to *S*. *cerevisiae* Gap1. Branches show bootstrap values (%), scale bar indicates amino acid substitutions per site. The fungal GAP proteins branched together with eleven putative GAP sequences of *F*. *fujikuroi* (orange box) (B) Expression analysis of putative GAP-encoding genes in *F*. *fujikuroi*. Total RNA was isolated from the wild-type and the *ΔAREA* and *ΔAREB* deletion mutants grown for 3 days in ICI liquid cultures with 6 mM (-Gln) or 60 mM (+Gln) glutamine as single nitrogen source and used for Northern blot analysis. 18S rRNA was visualized as loading control.

To infer the evolutionary relationship between the 19 candidates and the four references we calculated a phylogenetic tree and selected closely related proteins in extracting the members of the smallest subtree that contains all four known reference GAPs (Fig 1A). This resulted in a refined candidate set of eleven putative *F*. *fujikuroi* GAP proteins (FFUJ_01137, FFUJ_05331, FFUJ_ 08309, FFUJ_08705, FFUJ_08913, FFUJ_09118, FFUJ_09356, FFUJ_10091, FFUJ_11370, FFUJ_11387, FFUJ_11624).

We next determined whether the expression of these putative Gap1-like encoding genes is repressed under nitrogen sufficient conditions as it has been shown for Gap1 in *S*. *cerevisiae* [13,40]. The wild-type was grown for three days under known conditions for nitrogen deficiency and nitrogen sufficiency with 6 mM or 60 mM glutamine, respectively [41]. To further determine whether the two major nitrogen regulators, the GATA transcription factors AreA and AreB, are involved in regulation of Gap1-like *AAP* gene expression, the *F*. *fujikuroi* Δ*AREA* [42] and Δ*AREB* [41] mutants were grown under the same conditions. The expression of the eleven putative Gap1-encoding genes was analyzed by Northern blot analysis (Fig 1B). The expression of five of the AAP-encoding genes (*FFUJ_08309*, *FFUJ_08705*, *FFUJ_08913*, *FFUJ_09356* and *FFUJ_11370*) was too low to be detected under any of the tested conditions (data not shown). Four of the genes, *FFUJ_09118*, *FFUJ_05331*, *FFUJ_01137* and *FFUJ_11624*, were expressed only under nitrogen starvation conditions in the wild type and in the Δ*AREB* mutant, but not expressed in the Δ*AREA* mutant indicating that they are subject to nitrogen metabolite repression and are expressed in an AreA-dependent manner (Fig 1B). The expression of *FFUJ_01137* and *FFUJ_11624* is more elevated in the Δ*AREB* mutant than in the wild type under nitrogen limiting conditions indicating that AreB could act as a transcriptional repressor of these AAPs. The gene *FFUJ_10091* shows low expression under all conditions while the expression of *FFUJ_11387* depends on the presence of both AreA and AreB under nitrogen sufficient conditions.

In *S*. *cerevisiae*, the *GAP1* gene is sensitive to NCR, and its expression is activated by either the GATA transcription factor Gln3 or the GATA transcription factor Gat1 when grown under poor (proline) nitrogen conditions [43]. To determine whether the two nitrogen-repressed and AreA-dependent *GAP1*-like genes (*FFUJ_09118*, *FFUJ_05331*) share other analogies with the *S*. *cerevisiae GAP1*, we proceeded with heterologous functional expression in yeast. Full length cDNA fragments of these genes were cloned into the yeast expression vector yEX-C [39]. The vectors were transformed into *S*. *cerevisiae gap1Δ*dip5*Δ* mutant strain M4276 [44] and the *gap1Δssy1Δ* mutant strain M4238 [10]. Dip5 is the only yeast transporter capable of glutamate transport beside Gap1 [44], while Ssy1 is not an active amino acid transporter, but acts as amino acid sensor that mediates the transcriptional activation of several amino acid permeases [15]. Additionally, the vector yEX-PcGAP1, expressing the *PcGAP1* gene from *P*. *chrysogenum* [39], and yEX-11370 containing the non-expressed *F*. *fujikuroi* AAP gene FFUJ_11370 were transformed as controls.

**Fig 2:**
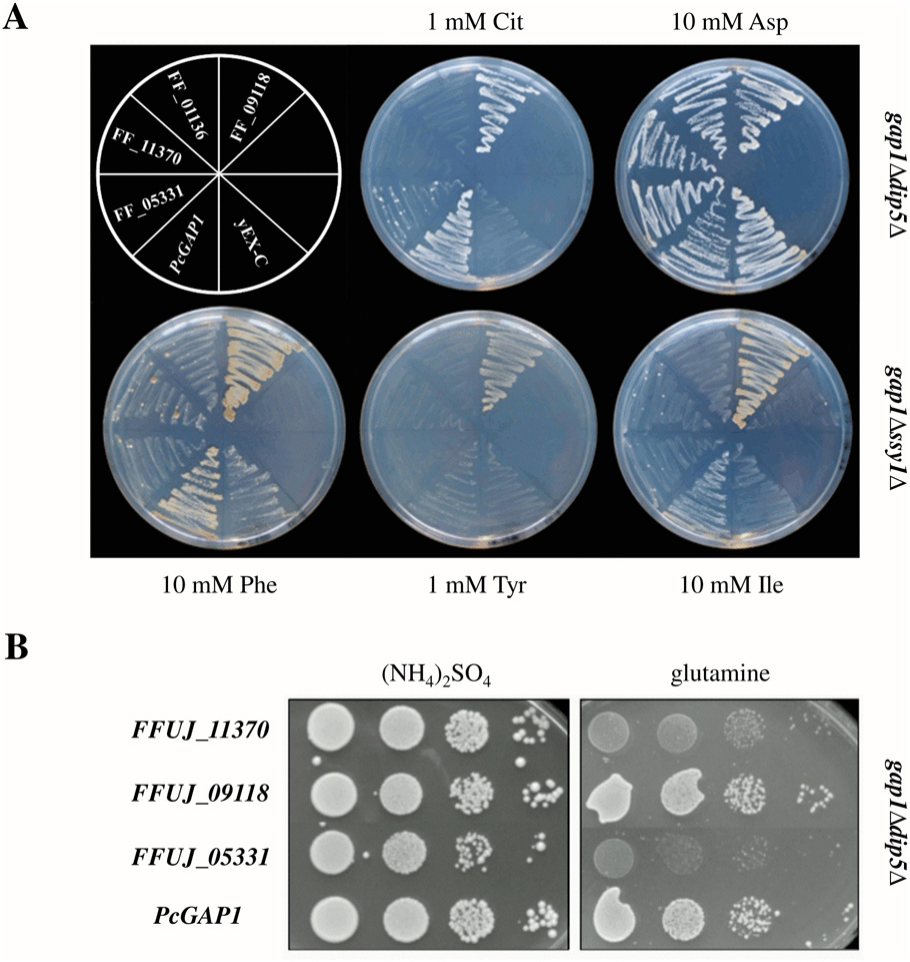
FFUJ_09118 is a functional homologue of *S*. *cerevisiae* Gap1. Nitrogen utilization growth assay of *S*. *cerevisiae* mutants *gap1Δdip5Δ* and *gap1Δssy1Δ* complemented with putative GAP-encoding genes of *F*. *fujikuroi* (*FFUJ_01137*, *FFUJ_05331*, *FFUJ_09118*, *FFUJ_11370*) and *PcGAP1* of *P*. *chrysogenum*. Fresh cells of tested yeast strains were adjusted to an optical density of 1 and (A) plated on yeast minimal agar with 1 mM citrullin (Cit), 10 mM aspartate (Asp), 10 mM phenylalanine (Phe), 1 mM tyrosin (Tyr) or 10 mM isoleucine (Ile) or (B) a series of 10-fold dilutions (left to right) were dropped on yeast minimal agar with 1 mM ammonium sulfate ((NH_4_)SO_4_) or glutamate as sole nitrogen source. Plates were incubated for 4 days at 30°C.

Yeast transformants were grown on minimal media each containing single amino acids as nitrogen source (Fig 2A, shown for FFUJ_05331, FFUJ_09118, FFUJ_01137, FFUJ_11370). First of all, we tested the ability of the potential AAPs to restore growth on minimal L-citrulline medium as transport of this amino acid is a peculiarity of the yeast Gap1 permease [45,46]. Of note, the *gap1Δdip5Δ* double mutant could only grow on citrulline when expressing the *P*. *chrysogenum* PcGAP1 or the *F*. *fujikuroi* AAP-encoding gene *FFUJ_09118*, suggesting that the latter *F*. *fujikuroi* permease acts as a Gap1 orthologue. Aspartic acid could obviously be utilized by other permeases than Dip5 and Gap1, as the double mutant displayed no growth defect with this amino acid. Furthermore, the *gap1Δssy1Δ* double mutant strain grew better on isoleucine and phenylalanine when expressing *PcGAP1* and the gene *FFUJ_09118*, indicating that both gene products are broad spectrum permeases that can complement many growth defects of this mutant. In addition, the capability to import tyrosine was completely lost in the *gap1Δssy1Δ* background, most likely due to the reported Ssy1-mediated activation of the main tyrosine transporter Bap2 [15]. Interestingly, only FFUJ_09118, but not PcGap1, was able to restore tyrosine uptake. To further address the specificity of this functional permease towards amino acids, strains were dropped in ten-fold dilution steps onto minimal media containing 1 mM ammonium sulfate as positive control, or 1 mM glutamate as sole N-source (Fig 2B). All of the tested yeast mutants (shown for FFUJ_09118, FFUJ_05331 and FFUJ_11370) grew well with ammonium as nitrogen source, while only the yeast transformants carrying PcGAP1 and FFUJ_09118 were able to efficiently utilize glutamate, suggesting that FFUJ_09118 encodes a functional *F*. *fujikuroi* permease further able to transport glutamate.

Taken together, these growth assays support that the gene *FFUJ_09118* encodes a likely Gap1 orthologue of *F*. *fujikuroi*. The gene will be referred to as *FfGAP1* in the following.

### Functional characterization of FfGap1 in *F*. *fujikuroi*

To test the substrate specificity and function of FfGap1, we created a deletion mutant in the *F*. *fujikuroi* wild-type strain. The deletion did not result in any growth defects on minimal medium, neither on glutamine, glutamate, proline, citrulline or tryptophane (data not shown). These data indicate that transport of these amino acids can be ensured by other permeases than *FfGAP1* and that the latter is not the sole pathway of citrulline uptake unlike the situation in yeast. The tested amino acids are obviously transported by one or more of the numerous additional AAPs present in the genome of *F*. *fujikuroi* [13].

**Fig 3:**
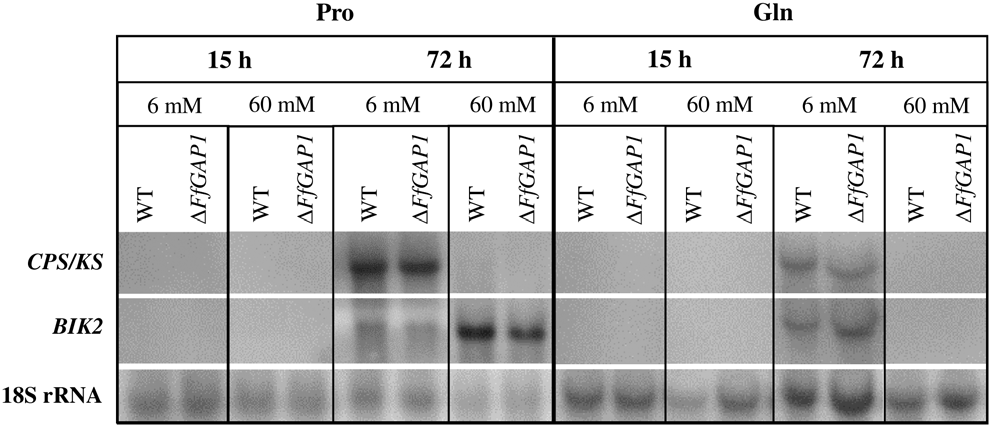
FfGap1 has no impact on nitrogen-mediated expression of SM biosynthesis genes. Total RNA was isolated from the wild-type and the Δ*FfGAP1* mutant grown for 3 days in liquid ICI medium with 6 mM (-Gln) or 60 mM (+Gln) glutamine as single nitrogen source and used for Northern blot analysis. The blot was hybridized with the *ent*-copalyldiphosphate/*ent*-kaurene synthase gene (*CPS/KS*) of the gibberellin and the monooxygenase gene (*BIK2*) of the bikaverin biosynthesis cluster. 18S rRNA was visualized as loading control.

Dual functions as nitrogen transporters and sensors have been described for several fungal nitrogen permeases, including the ammonium permeases Mep2 of *S*. *cerevisiae* [17,20] and MepB of *F*. *fujikuroi* [22], as well as the yeast Gap1 [16,47]. To investigate a possible dual function of FfGap1, we examined whether this permease plays a sensing role similarly to what was shown for MepB [22]. In this case the nitrogen-repressed SM genes would be highly expressed in the Δ*FfGAP1* mutant under nitrogen-sufficient conditions. We cultivated the *F*. *fujikuroi* wild-type and Δ*FfGAP1* mutant with low (6 mM) and high (60 mM) proline and glutamine levels for 15 h and 72 h. Expression of two SM biosynthesis genes, the *ent*-copalyldiphosphate/*ent-*kaurene-synthase gene (*CPS/KS*) of the GA and the monooxygenase gene (*BIK2*) of the bikaverin biosynthesis pathways, were analyzed by Northern blot analysis (Fig 3). The GA and bikaverin biosynthesis genes are subject to nitrogen metabolite repression in an AreA-dependent and AreA-independent manner, respectively [1,48]. In general, the observed expression patterns were similar in both the wild-type and Δ*FfGAP1* strains. At 15 h, when the fungus has not yet consumed the majority of the nitrogen source, the genes are not expressed. After 72 h the *CPS/KS* and *BIK2* genes are highly expressed under low, but not under high glutamine conditions as shown before [41,49,50]. However, with proline as nitrogen source *BIK2* is even stronger expressed at high compared to low proline concentrations while the GA gene is strongly repressed. These data underline the different nitrogen regulation mechanism for GA (AreA-dependent) and bikaverin (AreA-independent) genes.

Taken together, the nitrogen repression of two SM gene clusters is not released in the Δ*FfGAP1* mutant indicating that FfGap1 is not involved in regulating expression of these genes in contrast to the ammonium permease MepB [22].

### Nitrogen-dependent intracellular localization of FfGap1

**Fig 4:**
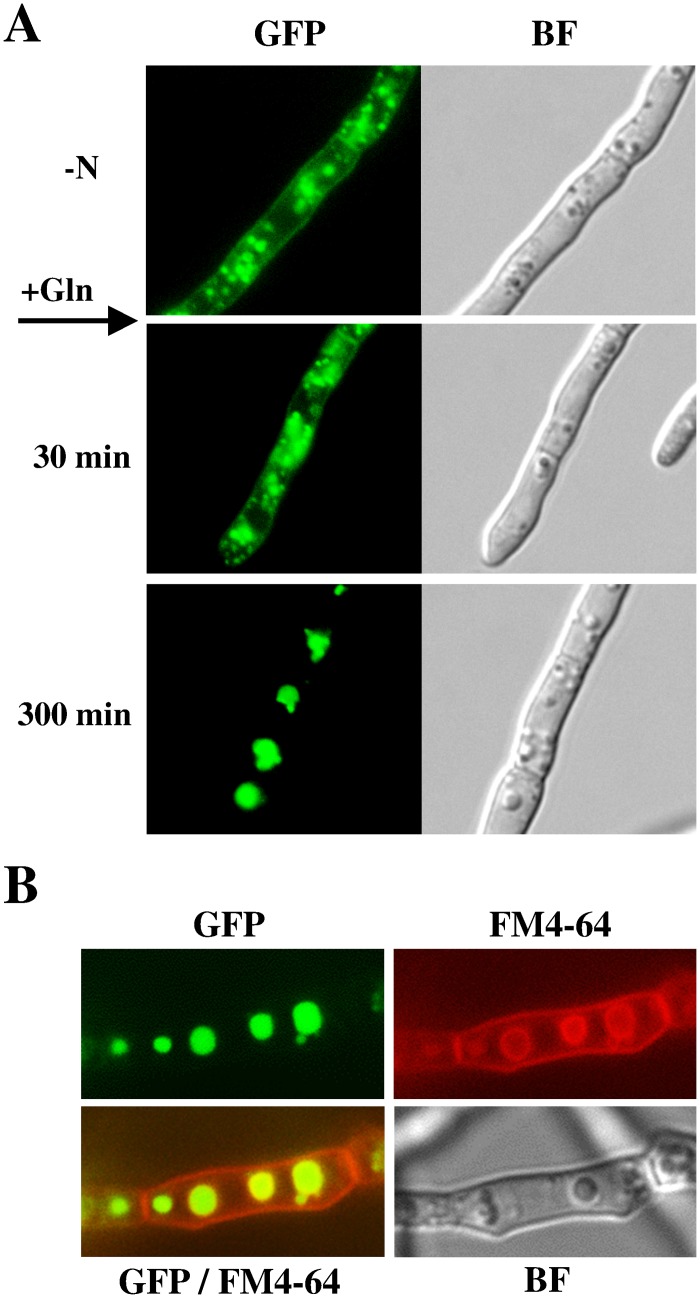
Subcellular localization of FfGap1 depends on nitrogen availability. *F*. *fujikuroi* wild type transformed with a FfGap1-GFP fusion construct was cultivated in liquid ICI medium with 6 mM glutamine for 48 h. (A) Cells were observed by fluorescence (GFP) and brightfield microscopy (BF) before (- N) and 30 min and 300 min after addition of 12 mM glutamine (+ Gln). (B) Cells were stained with FM4-64 and observed 60 min after addition of 12 mM Gln by fluorescence (FM4-64, GFP) and brightfield (BF) microscopy.

The *S*. *cerevisiae* Gap1 is actively transported to the plasma membrane, when no rich nitrogen source is available [27]. We next assessed whether FfGap1 could be similarly regulated by the quality of the nitrogen supply. To follow up the subcellular localization of FfGap1 under different nitrogen conditions, a FfGAP1-GFP fusion construct was generated and transformed into the Δ*FfGAP1* deletion strain. The correct integration of the full length *FfGAP1-GFP* construct at the *FfGAP1* locus was shown for several transformants by diagnostic PCR (data not shown). The wild-type and one of the FfGAP1-GFP strains were grown for 48 hours in liquid ICI medium under nitrogen-limiting (6 mM glutamine) conditions. At this time point, glutamine is exhausted and the fungus suffers already from nitrogen starvation [51]. Fluorescence microscopy revealed that the FfGap1-Gfp fusion protein was localized to the plasma membrane, but also in small cellular structures in the cytoplasm. We next assessed the impact of nitrogen re-supplementation on FfGap1 localization. After addition of 12 mM glutamine, the subcellular localization of FfGap1 was studied for a period of 5 hours (Fig 4A). At 30 min after addition of glutamine, membrane localization is notably weaker compared to the initial nitrogen starvation conditions. Furthermore, the size of fluorescent intracellular structures, was continuously increasing and small moving structures, probably endosomes, were observed. Five hours upon glutamine addition, the FfGap1-Gfp signal was completely absent at the plasma membrane and instead was concentrated in large, circular structures, probably the vacuoles. Exactly the same effects on FfGap1 localization were observed when proline was used as a nitrogen source instead of glutamine (data not shown). These data indicate that intracellular sorting of FfGap1 strictly depends on nitrogen availability as it is the case in *S*. *cervisiae*. However, in contrast to yeast where newly synthesized Gap1 is also delivered to the plasma membrane when cells are grown on poor substrates such as proline or urea as the sole nitrogen source [26], proline is recognized as a good nitrogen source in *F*. *fujikuroi* leading to FfGap1 sorting from the plasma membrane to the vacuoles.

To confirm that these fluorescent structures are indeed vacuoles, we performed FM 4–64 [*N*-(3-triethylammoniumpropyl)-4-(*p*-diethylaminophenyl-hexatrienyl) pyridinium dibromide] staining to compare the staining patterns of the dye with GFP fluorescence. FM 4–64 staining was previously shown to be specific for vacuolar membranes [52,53]. Indeed, FM 4–64 and the FfGap1-Gfp signal colocalized specifically at the large developed vacuoles (Fig 4B).

In summary, FfGap1 appeared to be localized mainly at the plasma membrane under nitrogen starvation conditions, while the protein was internalized and sorted to the lumen of the vacuole under nitrogen-sufficient conditions as it has been shown for the *S*. *cervisiae* Gap1.

### Identification of the Npr1 homologue of *F*. *fujikuroi*

After demonstrating that FfGap1 sorting is regulated by nitrogen availability, we next asked whether a Npr1-like protein kinase could be involved in such a process. So far, no Npr1-homologs are described in any filamentous fungus. A BLASTX search with the *S*. *cerevisiae* Npr1 protein sequence against the *F*. *fujikuroi* genome database [7] revealed three best hits (FFUJ_04942, FFUJ_02924 and FFUJ_05668), all possessing serine/threonine protein kinase InterPro motifs. FFUJ_04942 showed the highest amino acid identity to ScNpr1 (33.1%), while FFUJ_02924 and FFUJ_5669 displayed only 22.7% and 21.3% identity, respectively. While FFUJ_04942 (774 amino acids) and FFUJ_05668 (736 amino acids) are similar in size to the *S*. *cerevisiae* Npr1 protein (790 amino acids), FFUJ_02924 is much smaller than the yeast Npr1 (500 amino acids). The N-terminal region of the protein sequences is rich in serine residues, albeit not as rich as in the case of yeast Npr1 (S1 Fig) [25]. The three Npr1 homologues will be referred to as FfNpr1-1 (FFUJ_04942), FfNpr1-2 (FFUJ_02924) and FfNpr1-3 (FFUJ_05668) in the following. To determine if one of the three ORFs encodes a functional *F*. *fujikuroi* Npr1-analogue, full length cDNA clones of all three genes were cloned into the YEplac195-npr1-Term expression vector containing the *S*. *cerevisiae NPR1* promoter and terminator regions. The three resulting vectors and the *S*. *cerevisiae* native *NPR1* gene in the yeast expression vector pMV33 as a positive control, were transformed into the wild-type yeast strain 23344c and the *npr1Δ* yeast mutant [54]. Transformants were screened for growth on different nitrogen sources. It has been previously shown that the yeast *npr1Δ* mutant is not able to grow on several nitrogen sources because Npr1 plays essential but differing roles in regulating the activity of nitrogen permeases. While the inability of the *npr1Δ* mutant to grow on a specific substrate of Gap1 (e.g. citrulline, and to lesser extend tryptophan) is due to the role of the Npr1 kinase to oppose the ubiquitination and subsequent vacuolar degradation of Gap1 [23,26,27], the inability to grow on low ammonium and the reduced growth on some other nitrogen sources (e.g. proline) is additionally due to inefficient retrieval of catabolic ammonium escaping from the cells, a consequence of the Mep inactivity [18].

**Fig 5:**
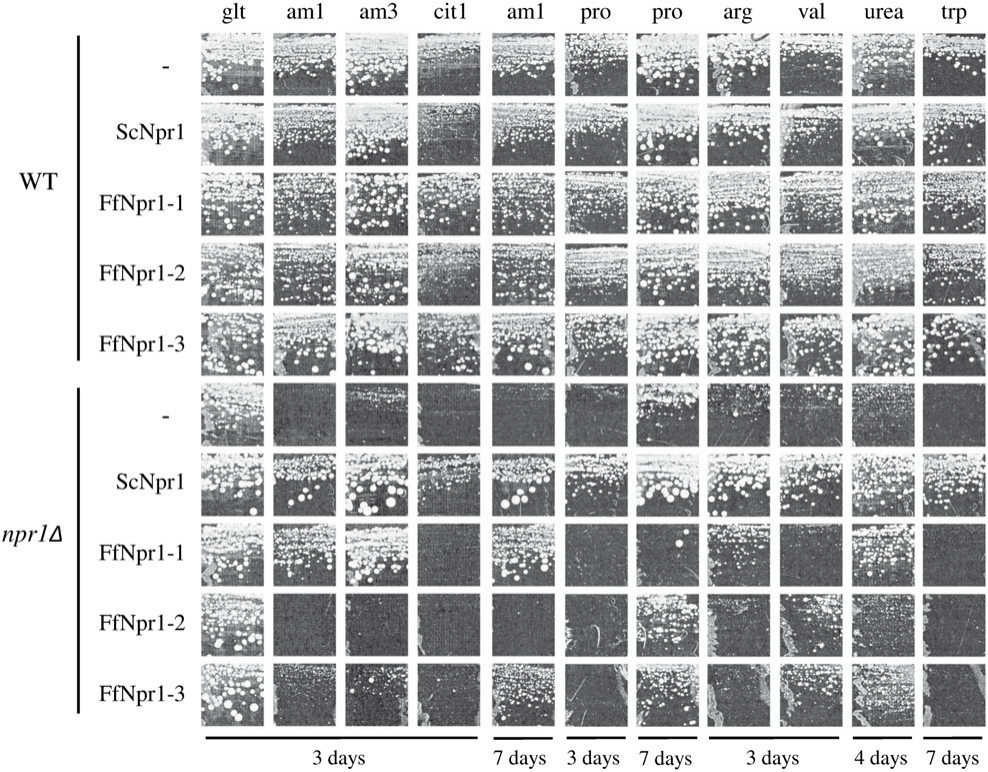
Cross-species complementation of the *S*. *cerevisiae npr1Δ* mutant with *F*. *fujiuroi* Npr1-like kinases leads to partial restoration of growth on different nitrogen sources. *S*. *cerevisiae* wild-type (WT) and *npr1Δ* mutant strains were transformed with *FfNPR1-1*, *FfNPR1-2*, *FfNPR1-3* and *ScNPR1*. Fresh cells of tested yeast strains were adjusted to an optical density of 1 and plated on yeast minimal agar with different nitrogen sources: 1 mM glutamate (glt), 1 mM ammonium sulfate (aml), 3 mM ammonium sulfate (am3), 1 mM citrulline (cit1), 1 mM proline (pro), 1 mM arginine (arg), 1 mM valine (val), 1 mM urea, 1 mM tryptophane (trp). Plates were incubated for 3 to 7 days at 30°C.

To compare the growth behavior of the yeast *npr1Δ* mutant with that of the transformants, all strains were plated on minimal medium containing different nitrogen sources (Fig 5). The wild-type was able to efficiently use all supplied nitrogen sources, while the yeast *npr1Δ* mutant did not or only weakly grow on all nitrogen sources except glutamate. Complementation of *S*. *cerevisiae* with the native *NPR1* gene fully restored growth of the *npr1Δ* yeast mutant on all nitrogen sources. Although none of the three *F*. *fujikuroi NPR1*-like genes was able to restore growth on all nitrogen sources, they were intriguingly able to mediate growth on specific nitrogen sources, respectively. FfNpr1-1 mediated growth on ammonium, urea and partially on arginine. FfNpr1-3 seemed to slightly improve growth on valine and, like FfNpr1-1, on urea while growth on ammonium was only partially restored after 7 days of incubation. FfNpr1-2 was only able to slightly support growth on valine.

**Fig 6:**
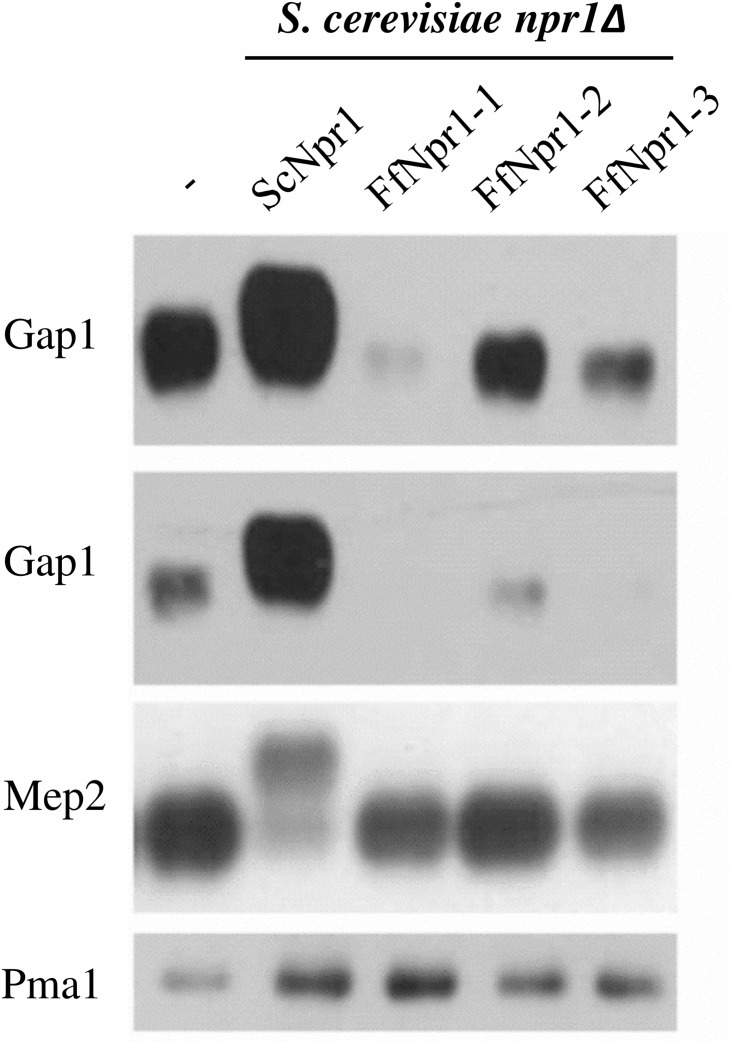
*F*. *fujikuroi* Npr1-like kinases do not have an impact on Gap1 stability in yeast. The *S*. *cerevisiae npr1Δ* mutant and *npr1Δ* transformed with *FfNPR1-1*, *FfNPR1-2*, *FfNPR1-3* and *ScNPR1* were cultivated in liquid minimal medium with low amounts of urea as sole nitrogen source. Membrane enriched protein extracts were used for Western blot analysis. Specific antibodies were used to detect Gap1 and the ammonium permease Mep2. Pma1 was detected as protein loading control.

The absence of growth restoration on citrulline indicates that none of the potential Npr1-like proteins from *Fusarium* can re-establish yeast Gap1 activity. To further examine if one of the three FfNpr1-like kinases has an impact on Gap1 stability in yeast, a Western analysis was performed with membrane enriched protein extracts of the *npr1Δ* strain and *npr1Δ* strains transformed with *F*. *fujikuroi NPR1* homologues. All strains were grown with urea which is a non-preferred N-source like proline (Fig 6). Hybridization with a Gap1 antibody revealed a stronger abundance of the permease in the *npr1Δ* cells expressing yeast ScNpr1 compared to *npr1Δ* cells transformed with an empty vector. None of the Npr1-like proteins enabled stabilization of Gap1. The *npr1Δ* cells transformed with *FfNPR1-1* showed a reduced Gap1 signal compared to the *npr1Δ* cells transformed with an empty vector suggesting that the heterologous FfNpr1 kinase could somehow favor the degradation of Gap1. This could explain the apparent loss of proline utilization observed upon *FfNPR1-1* expression. FfNpr1-2 and FfNpr1-3 expression were accompanied with a similar effect on the Gap1 detected levels, although to a lesser extent.

Yeast Npr1 was recently shown to control the Mep2 ammonium transport activity by mediating phosphorylation of the permease [30]. Mep2 thereby appears as a double band on SDS-page, the slow-migrating form corresponding to the phosphorylated and active Mep2 permease. Our western blot analysis shows that in contrast to yeast Npr1, none of the three potential Npr1-like proteins from *Fusarium* restore the phosphorylation of Mep2. We thereby conclude that growth on ammonium of *npr1Δ* cells expressing FfNpr1-1 probably relies on activation of Mep1 and/or Mep3 but not on Mep2.

Taken together, at least two of the 3 Npr1-like *F*. *fujikuroi* kinases (FfNpr1-1 and FfNpr1-3) appear to have an impact on the functionality of different permeases of nitrogen sources in *S*. *cerevisiae*. The targets of the different FfNpr kinases seem to overlap in some cases. Our data suggest that the functions of yeast Npr1 could be split among different kinase proteins in *F*. *fujikuroi*, mainly FfNpr1-1 and FfNpr1-3. However, none of the tested *Fusarium* Npr1-like proteins is able to stabilize and restore yeast Gap1 function.

### FfNpr kinases only slightly influence nitrogen utilization in *F*. *fujikuroi*

**Fig 7:**
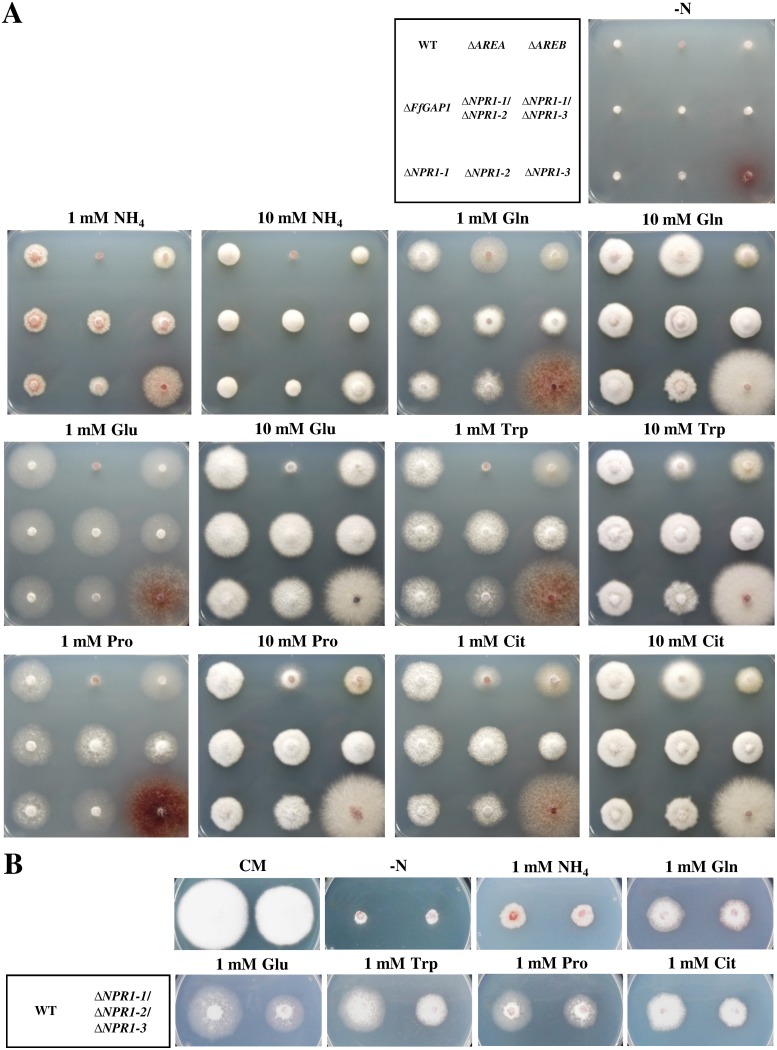
Growth assay of *ΔFfGAP1* and *ΔFfNPR1-1*, ΔFfNPR1-2 and *ΔFfNPR1-3* single, double and triple mutants on different nitrogen sources. Strains were grown on solid ICI minimal medium with either no nitrogen (-N) or the indicated concentrations of various nitrogen sources (A, B) or on CM complete medium (B) at 28°C for 4 days.

To elucidate a potential role of the three FfNpr1 kinases in *F*. *fujikuroi*, *FfNPR1-1*, *FfNPR1-2* and *FfNPR1-3* deletion mutants were generated (for details, see Materials and Methods section). Homologous *in locus* integration of the gene replacement fragments was confirmed by Southern Blot or diagnostic PCR (S2 Fig). Additionally, we generated ΔΔ*FfNPR1-1/FfNPR1-2* and ΔΔ*FfNPR1-1/FfNPR1-3* double deletion mutants. The growth of the wild-type, Δ*FfGAP1*, and the single and double *NPR1* deletion strains was examined after 4 days incubation on minimal agar media with different nitrogen sources at two concentrations (1 and 10 mM). Each growth assay was done with two independent transformants for each deletion experiment. As they showed an identical growth behavior, plate assays are shown for one transformant each (Fig 7A). For comparison, Δ*AREA* and Δ*AREB* mutants which have lost one of the two global nitrogen regulators, the GATA transcription factors AreA or AreB [13], were included into these growth assays.

While the Δ*AREA* and Δ*AREB* mutants revealed the well known growth defects on some nitrogen sources [41], the *FfNPR1* mutants did not show significant growth defects compared to the wild-type on all tested media. Surprisingly, the *FfNPR1-3* mutant showed a significantly increased colony diameter compared to the wild-type on all nitrogen sources, including ammonium. The colonies appeared less dense and sparse aerial hyphae. In addition, the Δ*FfNPR1-3* mutant produced a red pigment under low nitrogen conditions (Fig 7A). However, this distinctive phenotype was not shared with the *FfNPR1-1*/*FfNPR1-3* double mutant, which showed a morphology similar to Δ*FfNPR1-1* and the wild-type. Complementation of the Δ*FfNPR1-3* mutant (Δ*FfNPR1-3* ^*C*^ *)* restored the wild-type growth on all tested amino acids, proving that the observed phenotype is caused by the *NPR1-3* deletion (S3 Fig). Despite the different morphology of the Δ*FfNPR1-3* mutant, the actual biomass produced on solid and liquid minimal media (1 mM glutamine) was the same for the wild-type and the Δ*FfNPR1-3* and ΔΔ*FfNPR1-1/FfNPR1-3* mutants (data not shown).

As none of the single and double mutants revealed a significant growth defect on one of the tested nitrogen sources, we generated ΔΔΔ*FfNPR1-1/FfNPR1-2/FfNPR1-3* triple deletion mutants and performed plate assays. Although these triple mutants showed a slightly reduced growth rate on all tested media compared to the wild-type, they still were able to utilize all nitrogenous compounds for growth (Fig 7B) suggesting that it is not attributed to a specific defect in amino acid uptake.

Summarizing, the *NPR1* deletion strains showed no significant growth defects on various amino acids, in contrast to the global nitrogen regulator mutants Δ*AREA* and Δ*AREB*. These data suggest that the tested FfNpr1 kinases have no major and global impact on the activity of amino acid permeases in *F*. *fujikuroi*.

### FfNpr kinases of *F*. *fujikuroi* do not influence the nitrogen dependent sorting of FfGap1

**Fig 8:**
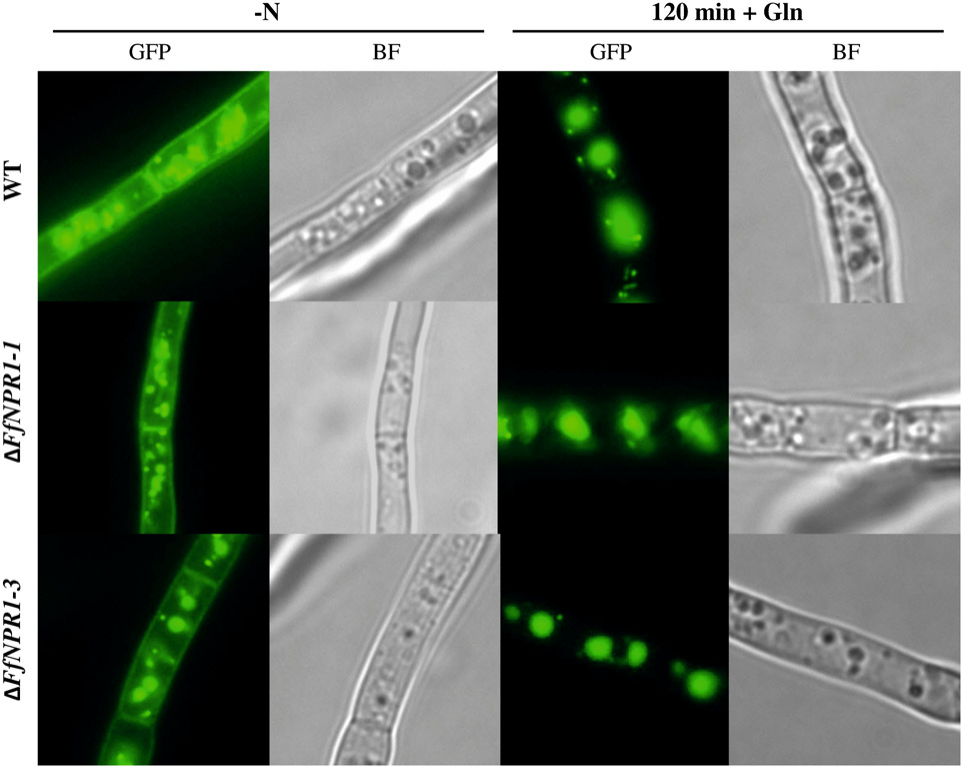
Npr1-like kinases do not influence intracellular sorting of FfGap1. *F*. *fujikuroi* wild-type and Δ*FfNPR1-1* and Δ*FfNPR1-3* mutants, all transformed with a FfGap1-GFP fusion construct, were cultivated in liquid ICI medium with 6 mM glutamine for 48 h. Cells were observed by fluorescence (GFP) and brightfield microscopy (BF) before (- N) and 120 min after addition of 12 mM glutamine (+ Gln).

As shown above, the sorting of FfGap1 depends on nitrogen availability. To assess whether any of FfNpr kinases affect this sorting process, we transformed the Δ*NPR1-1* and Δ*NPR1-3* mutant strains with the plasmid pFfGAP1-GFP. The generated transformants were selected for *in locus* integrations of the *FfGAP1-GFP* construct. The resulting Δ*NPR1-1*/*FfGAP1-GFP* and Δ*NPR1-3*/*FfGAP1-GFP* transformant strains and the wild type carrying the *FfGAP1-GFP* construct were grown for 1 day under nitrogen limiting conditions (3 mM glutamine), and the cultures were monitored by fluorescence microscopy before and 120 minutes after the addition of 12 mM glutamine (Fig 8).

As in the wild-type, both *FfNPR1* mutants displayed an abundance of FfGap1-Gfp in the plasma membrane during starvation, while the fusion protein was completely sorted to the vacuoles 120 minutes after addition of glutamine, indicating that the single deletion of FfNpr1-1 and FfNpr1-3 does not affect nitrogen-dependent sorting of FfGap1.

Similarly, the addition of nitrate or ammonium resulted in continuous enrichment of the fluorescence signal in the vacuoles in all strains (S4A, S4B and S4C Fig). However, with nitrate it took longer to transport FfGap1 to the vacuoles in both the wild-type and the mutant backgrounds, compared to glutamine and ammonium. This delay is probably due to the fact that nitrate has to be converted first to ammonium and glutamine to be used by the fungus.

Summarizing, these microscopic observations indicate that intracellular sorting of FfGap1 in response to nitrogen does not exclusively depend on the activity of the tested Npr1-like protein kinases in *F*. *fujikuroi*. Nevertheless, sorting of FfGap1 depends on the nitrogen availability.

### Sorting of FfGap1 depends on conserved lysine residues

In *S*. *cerevisiae* it has been shown that endocytosis and degradation of Gap1 upon addition of the preferred nitrogen source ammonium is triggered by ubiquitination of N-terminal lysine residues at positions 9 and 16. Npr1 prevents the ubiquitination of Gap1 at these lysine residues during growth on non-preferred nitrogen sources [26,27,55,56].

**Fig 9:**
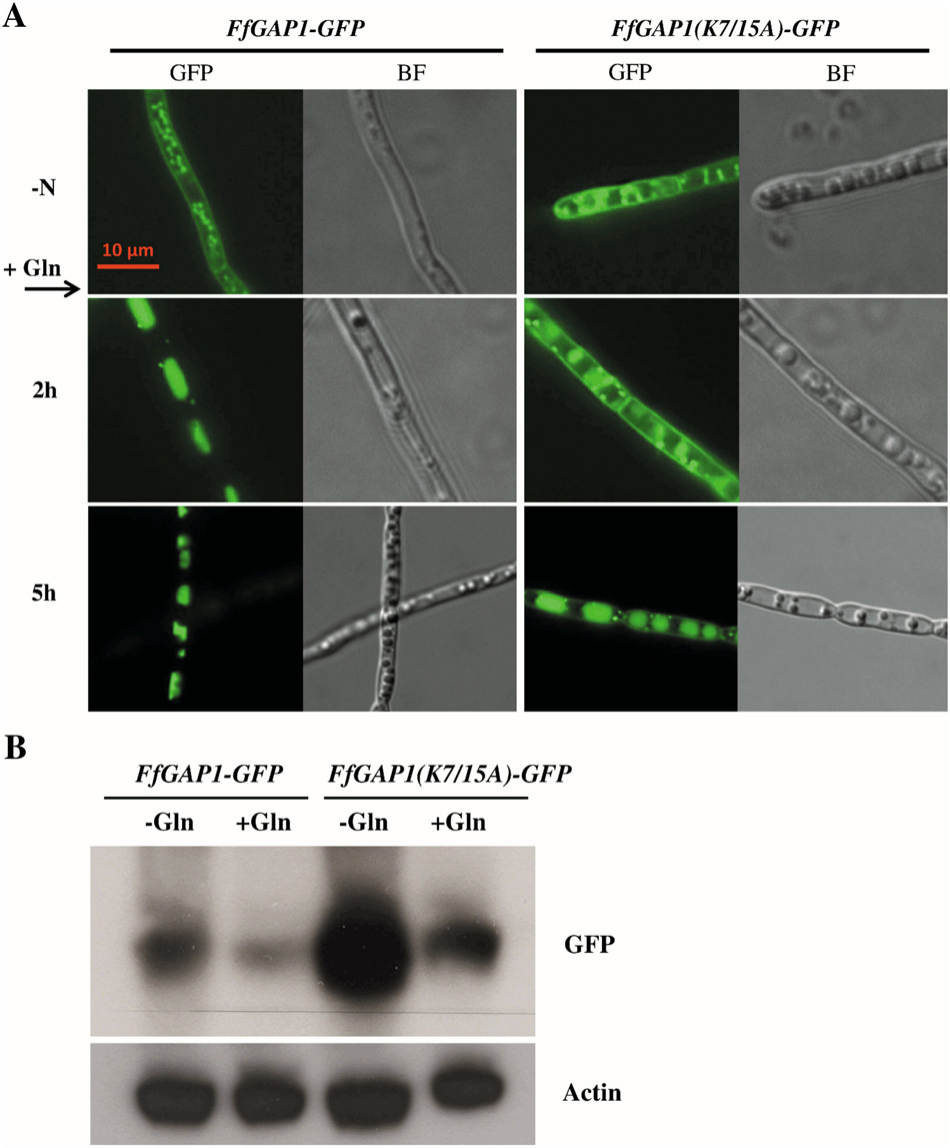
Conserved lysine residues influence sorting and protein stability of FfGap1. *F*. *fujikuroi* wild-type transformed with the FfGap1-Gfp or the FfGap1(K7/15A)-Gfp fusion construct was cultivated in liquid ICI medium with 6 mM glutamine for 48 h. (A) Cells were observed by fluorescence (GFP) and brightfield microscopy (BF) before (- N) and 2 h and 5 h after addition of 12 mM glutamine (+ Gln). (B) Western blot analysis of protein extracts before (- N) and 2h after addition of 12 mM glutamine (+ Gln). Hybridization with an anti-actin (actin) antibody was used as protein loading control.

To examine whether ubiquitination might be involved in FfGap1 sorting, we compared the sequence of the Gap1 proteins of *S*. *cerevisiae* and *F*. *fujikuroi* and found two potential ubiquitination targets at position K7 and K15 of FfGap1. Point mutations were introduced into the respective codons to exchange both lysine residues for alanine (K7A; K15A). The mutated and wild-type *FfGAP1-GFP* fusion constructs were transformed into the Δ*FfGAP1* mutant. The resulting strains expressing *FfGAP1(K7/15A)-GFP* and *FfGAP1-GFP* (wild-type FfGAP1), respectively, were cultivated under nitrogen starvation conditions for 48 hours. The subcellular localization of FfGap1-Gfp and FfGap1(K7/15A)-Gfp was examined by fluorescence microscopy before and up to 5 hours after the addition of 12 mM glutamine. Gfp signals of both the wild-type and the mutated FfGap1 were detected at the plasma membrane and in intracellular structures under nitrogen starvation conditions (Fig 9A). Two hours after addition of glutamine, the wild-type FfGap1 was totally absent from the membranes and localized in the lumen of vacuoles, as shown before (Fig 4). In contrast, the mutated FfGap1(K7/15A) fusion protein was still localized at the membranes at this time point, and traces of the mutated protein were visible at the membrane even five hours after addition of glutamine. Longer incubation (6 hours and more) resulted in a complete sorting of the mutated Gap1-Gfp proteins to the vacuoles (data not shown). These data indicate that the substitution of the lysine residues at position 7 and 15 of FfGap1 has an impact on the duration of membrane localization after adding nitrogen. However, the general nitrogen-dependent sorting to the vacuoles was significantly delayed but not fully abolished.

To test whether the mutations have an effect on general protein stability and abundance, Western blot analyses were performed using an anti-GFP antibody. Strains carrying FfGAP1-GFP or the mutated FfGAP1(K7/15A)-GFP were cultivated with low nitrogen for 72 hours and harvested before and 2 hours after the addition of 12 mM glutamine (Fig 9B). The amount of the wild-type protein drastically decreased after addition of glutamine, indicating a nitrogen-dependent degradation of the protein. The amount of the mutated protein was significantly higher compared to the wild-type under both conditions what fits well to the observed delayed degradation process of FfGap1(K7/15)-Gfp after addition of glutamine. These data indicate that the lysine residues K7 and K15 are involved in nitrogen-dependent degradation, though additional factors might also intervene in the protein stability and sorting.

## Discussion

Recently, it has been shown that in *F*. *fujikuroi*, most of the 45 SM gene clusters are regulated by nitrogen availability [7]. Some of them are targets of the GATA transcription factors AreA and/or AreB [5,6,41,49]. In addition to SM biosynthetic genes, a set of nitrogen permease-encoding genes, such as the three ammonium transporter-encoding genes *MEPA*, *MEPB*, and *MEPC* or the peptide transporter-encoding gene *MTD1* are subject to AreA-mediated nitrogen metabolite repression in *F*. *fujikuroi* [22,50]. Studies on *S*. *cerevisiae* reported that two nitrogen permeases, the ammonium transporter Mep2 [17,18,19] and the general ammonium permease Gap1 [16,57], also function as receptors for rapid activation of the protein kinase A (PKA) pathway upon addition of their substrate [58]. It is not known whether a similar transceptor function of Gap1 homologues and a functional link to the PKA pathway exist in filamentous fungi. In *F*. *fujikuroi*, a potential dual function as transporter and receptor has been shown so far only for the ammonium permease MepB [22]. Its deletion resulted in upregulation of otherwise nitrogen-repressed genes, e.g. the GA and bikaverin biosynthesis genes, under ammonium-sufficient conditions [22]. However, a potential dual role of Gap1 homologues as permeases and receptors have never been shown in any filamentous fungus. It is also not known whether the stability of Gap1 homologues is regulated by a Npr1-like protein kinase as it has been shown in yeast [36,46].

In this work, we identified and characterized a Gap1-homologue in *F*. *fujikuroi* and studied its regulation on transcriptional and protein levels as well as the impact of potential Npr1-like protein kinases on FfGap1 stability and subcellular localization.

### Identification and function of FfGap1

The analysis of the recently sequenced genome of *F*. *fujikuroi* [7] revealed 99 potential AAP proteins with the characteristic AAP protein domains. This is a significantly higher number compared to 20, 27, and only 19 identified AAPs in the genomes of *S*. *cerevisiae*, *Candida albicans* and *Aspergillus nidulans*, respectively [59,60]. Among these genes, only *FFUJ_09118* and *FFUJ_05331* were highly expressed under nitrogen-limiting conditions in an AreA-dependent manner similar to the Gln3-dependent expression of the yeast *GAP1* gene [40]. Yeast complementation experiments were consistent with FFUJ_09118 corresponding to a functional analogue of yeast Gap1. However, deletion of *FfGAP1* in *F*. *fujikuroi* did not result in a distinct phenotype for a specific amino acid suggesting that the loss of FfGap1 function can be compensated by the presence of numerous additional transporters. In addition, we cannot exclude that among the 99 potential AAPs more Gap1-like proteins exist with a broad spectrum of transported amino acids similarly to *Candida albicans* [59].

Deletion of *FfGAP1* did also not reveal any alterations in expression pattern of nitrogen-repressed SM-genes suggesting that FfGap1 does not possess an additional regulatory role as a nitrogen-sensing transceptor, which was described for Gap1 and several more nutrient transporters in *S*. *cerevisiae* [16,57,61] or the ammonium permease MepB in *F*. *fujikuroi* [22].

### FfGap1-sorting depends on nitrogen availability

After demonstrating that the expression of *FfGAP1* is regulated in an NCR-sensitive and AreA-dependent manner, we were intrigued by the possibility that sorting of FfGap1 to the plasma membrane might, like in yeast, depend on the nitrogen status of the cell. Fluorescence microscopy revealed abundance of FfGap1-Gfp at the plasma membrane during nitrogen starvation and migration of the fusion protein to the lumen of vacuoles. The active translocation of FfGap1 to the organelles has been observed in the time course as moving, fluorescence emitting structures.

The transport of FfGap1-Gfp to the vacuoles upon glutamine addition is similar to the findings in yeast [14,62]. However, in contrast to yeast, addition of proline also led to rapid sorting of FfGap1-Gfp to the vacuoles while nitrate-mediated delocalization of FfGap1-Gfp took longer. This delayed sorting could be explained by the time needed for converting nitrate to glutamine through nitrite and ammonium. Previously we have shown that repression of NCR target genes, e.g. the GA and bikaverin biosynthesis genes, by nitrate takes also much longer than with glutamine and ammonium [41].

Ammonium ions also have a strong negative impact on the plasma membrane localization of FfGap1. This is in contrast to the studies of Roberg and coworkers [63], who found a stimulatory effect of ammonium on Gap1 activity in *S*. *cerevisiae*. Contradictory to the findings by Roberg, other authors described the inactivation of Gap1 upon addition of ammonium to poor nitrogen sources, data that supports our present findings [64,65,66]. The apparent differences might be elucidated by different growth conditions and strain backgrounds used by the different researchers.

It is remarkable that cytoplasmic FfGap1-Gfp was detectable at all times, indicating constant sorting of FfGap1. The visible smaller intracellular structures carrying FfGap1-Gfp during nitrogen starvation may be part of the *trans*-Golgi network or the multivesicular endosome, which both participate in ubiquitination-dependent Gap1 sorting in yeast [27,61, 67]. The dynamic regulation of Gap1 sorting in *S*. *cerevisiae* allows the cell to rapidly enhance the import of amino acids when internal amino acid levels decrease. On the other hand, strict regulation of Gap1 sorting avoids excessive accumulation of amino acids that were found to be harmful [63,64]. A similar process might be important in *F*. *fujikuroi* to respond quickly to changing nitrogen conditions.

### Intracellular sorting and stability of FfGap1 is influenced by conserved lysine residues

In yeast it was shown that a set of enzymes facilitate ubiquitination of specific lysine residues at position 9 and 16 of ScGap1, which then leads to endocytosis and subsequent degradation in the vacuole. Mutation of these residues resulted in a stable Gap1 protein at the plasma membrane, even when favored nitrogen sources like ammonium are available [27].

In order to investigate a similar mechanism in *F*. *fujikuroi*, we substituted the conserved lysine residues at position 7 and 15 of FfGap1 by alanine. Localization experiments revealed that the mutated protein FfGap1(K7/15A) remains much longer at the plasma membrane after addition of glutamine. In addition, these point mutations resulted in overall increased stability compared to the wild type-protein. According to the yeast-model, it is most likely that FfGap1 is, in a similar way, regulated on a post-translational level by ubiquitination of the lysine residues. However, the actual involvement of ubiquitination has yet to be shown experimentally by monitoring the grade of ubiquitination of FfGap1 and identification of potential proteins that enable ubiquitination. Potential candidates for such proteins could be homologues of the *S*. *cerevisiae* ubiquitin-protein ligase Rsp5 or the Bul1 and Bul2 adaptor proteins, which are the key components of Gap1-ubiquitination in yeast [28,29].

Despite these similarities, the loss of the conserved residues K7 and K15 did not result in complete membrane-stabilization of FfGap1 under nitrogen surplus as it has been shown for *S*. *cerevisiae*. Furthermore, although the overall stability of the mutated protein was greatly increased in comparison to the wild-type, it was still subject to a nitrogen-induced degradation when glutamine was added, though at a much slower rate. These data suggest that there are either more lysine residues which are targets of ubiquitination, and/or that additional factors might be involved in nitrogen-induced destabilization of the permease. Finding the right target for the precisely regulated ubiquitination will be an interesting aim of future studies.

### *F*. *fujikuroi* Npr1 homologues slightly influence nitrogen utilization but have no apparent influence on FfGap1 sorting

In yeast, the serine/threonine protein kinase ScNpr1 positively influences the stable localization of Gap1 in the membrane during poor nitrogen supply [27]. The genome of *F*. *fujikuroi* contains three genes coding for Npr1-like proteins, and complementation of the *ScNPR1* yeast mutant with these three candidate genes overcome some, but not all, of the Δ*Scnpr1* growth defects. FfNpr1-1 provided more Npr1-like effects than the other two FfNpr kinases, especially compared to FfNpr1-2. The protein sequence of FfNpr1-2 is significantly shorter than the yeast Npr1 and the other two *F*. *fujikuroi* homologues and contains less serine residues at the N-terminus (S1 Fig), which could explain the low functional conservation of both proteins. The surprising and complete inability of the *FfNPR1-1*-complemented yeast Δ*ScNPR1* mutant to grow on proline indicates that FfNpr1-1 seems to inhibit the uptake of proline, which is mediated by the two nitrogen-regulated permeases Gap1 and Put4 as well as two other AAPs, Gnp1 and Agp1 [68,69].

Furthermore, absence of growth of FfNpr1-expressing cells on citrulline and tryptophane reveals that FfNpr1 did neither restore yeast Gap1 functions nor stabilize the protein, indicating significant differences between ScNpr1 and FfNpr1-1. The results of Western analysis confirmed our assumption that FfNpr1-1 is not able to reconstitute Gap1 activity. Only yeast cells transformed with the native *ScNpr1* gene were able to prevent degradation of Gap1 in the *S*. *cerevisiae npr1Δ* mutant when incubated with urea. Interestingly, activity of FfNpr1-1 in the Δ*ScNPR1* background seems to enhance the degradation process of Gap1 even more than in the deletion mutant. This could explain the complete inability of the FfNpr1-1-complemented yeast strain to grow on proline, assuming that a similar effect acts on Put4. FfNpr1-2- and FfNpr1-3-complemented yeast strains also displayed a weaker abundance of Gap1. Nevertheless, the decline of Gap1 is much more severe in the FfNpr1 strain, suggesting major regulatory defects caused by FfNpr1-1.

Single, double and triple deletions of *FfNPR1* genes in *F*. *fujikuroi* revealed no major defects in utilization of certain nitrogen sources. Deletion of *FfNPR1-3* resulted in the most severe effect. The growth morphology of the mutant with an enhanced colony diameter but reduced aerial mycelium is reminiscent of the growth of the wild-type on poor nitrogen sources or under overall low nutrient availability. This indicates that the utilization of all tested nitrogen sources might be slightly impaired in the case of Δ*FfNPR1-3*. Complementation of the mutant with the *FfNPR1-3* gene copy fully restored the wild type phenotype. However, we have no explanation why this phenotype is not shared by the ΔΔ*FfNPR1-1*/*FfNPR1-3* double or ΔΔΔ*FfNPR1-1*/*FfNPR1-2*/*FfNPR1-3* triple mutant. Currently, the role of all three Npr1 homologues in *F*. *fujkuroi* is unknown and needs further investigation. It is also not known by which mechanism FfNpr1-3 affects utilization of nitrogen sources in *F*. *fujikuroi* and upon heterologous expression in *S*. *cerevisiae*. However, we were able to demonstrate that, unlike in yeast, the tested Npr1 homologues in *F*. *fujikuroi* are not exclusively involved in regulating the sorting and stability of FfGap1. These results are in accordance with the inability of the FfNpr kinases to restore Gap1 activity and stability in the *npr1Δ* yeast mutant.

In summary, we identified a functional homologue of the *S*. *cerevisae* Gap1 among the 99 potential AAPs in *F*. *fujikuroi*. FfGap1 is able to restore growth defects of the yeast *gap1* mutant. Subcellular localization of FfGap1 strictly depends on nitrogen availability but not on the presence of Npr1-like proteins in *F*. *fujikuroi*. Furthermore, two conserved lysine residues (K9, K15) are probably involved in ubiquitination-mediated degradation of FfGap1.

## Material and Methods

### Fungal strains and culture conditions

The following *F*. *fujikuroi* strains were employed: wild-type strain IMI58289 (Commonwealth Mycological Institute, Kew, UK), Δ*AREA*-T19 [42] and Δ*AREB*-T2.1 [41].

For submerse culture experiments *F*. *fujikuroi* strains were first grown for 72 h at 28°C in 300 ml Erlenmeyer flasks with 100 ml Darken medium [70] on a rotary shaker. 500 μl of this culture were then used to inoculate 100 ml of ICI (Imperial Chemical Industries, UK) media [71] containing either 6 mM glutamine or 60 mM glutamine. Growth proceeded for 1–3 days on a rotary shaker at 28°C in the dark. For DNA isolation and protoplasting, *F*. *fujikuroi* strains were incubated in 100 ml modified ICI medium (Imperial Chemical Industries Ltd., UK) [71] containing 10 g/l fructose instead of glucose and 0,5 g/l (NH_4_)_2_SO_4_ as nitrogen source at 28°C on a rotary shaker at 200 rpm for 3 days and 18 h, respectively. For RNA isolation, the fungal strains were grown in synthetic ICI medium with 6 mM glutamine, 30 mM glutamine or 12 mM NaNO_3_ for 3 days. After this time, the mycelia were harvested, washed with deionized water, and flash frozen with liquid nitrogen prior to lyophilization. For fluorescence microscopy the fungal strains were grown in synthetic ICI medium with 6 mM glutamine for 3 days or with 3 mM glutamine for 1 day. After this time the strains were examined via microscopy before and after addition of glutamine, (NH_4_)_2_SO_4_ or NaNO_3_ up to a concentration of 12 mM or 24 mM. To compare the growth of the *NPR1* deletion mutants with that of the *F*. *fujikuroi* wild-type, the strains were grown on solidified (15 g/l agar) ICI-media containing different nitrogen sources as specified in the text. Plates were incubated at 28°C in the dark and pictures of the plates were taken at different time points.

The following *S*. *cerevisiae* strains were employed for complementation tests with *F*. *fujikuroi AAP* genes: *gap1Δdip5Δ* mutant strain M4276 (*MAT* **a** *ura3* Δ*GAP1*; isogenic to strain S288C) [44], and *gap1Δssy1Δ* mutant strain M4238 (*MATa ura3* Δ*GAP1* Δ*SSY1*; isogenic to strain S288C) [10]. For complementation with *F*. *fujikuroi NPR* genes, the *S*. *cerevisiae* wild-type strain 23344c and the Δ*ScNPR1* strain 30788a [27] have been used. Yeast cells of strain M4276 and M4238 were grown either in minimal medium without ammonium sulfate (10 g/l succinic acid, 6 g/l NaOH, 1.7 g/l yeast nitrogen base (Difco, Lawrence, USA) and 20 g/l D-glucose) or with addition of nitrogen sources as indicated in the text. Yeast cells of strain 23344c and 30788a were grown in minimal buffered (pH 6.1) medium with 3% glucose as the carbon source [72]. To this medium, nitrogen sources were added as required by the experiment and as specified in the text.

For yeast recombination cloning *S*. *cerevisiae* strain FGSC9721/FY834 (*MATa his3Δ200 ura3-52 leu2Δ1 lys2Δ202 trplΔ63*) [73] was cultivated in 5 ml liquid YPD (pH 5.8, 10 g/l yeast extract, 20 g/l Bacto-Trypton (Difco), 20 g/l glucose) overnight at 200 rpm and 30°C. The overnight culture was used to inoculate 50 ml liquid YPD and incubated at 200 rpm and 30°C for 4 to 6 hours until it reached an OD_600nm_ of around 1. The harvested yeast cells were further used for yeast recombination cloning [74,75].

### Yeast growth assays

The growth of *S*. *cerevisiae* strains transformed with Yep352 (empty plasmid), pScGAP1 (positive control) and plasmids carrying one of the tested *F*. *fujikuroi* AAP-encoding genes was tested on plates with minimal medium supplemented with ammonium sulfate or amino acids as described [59]. For growth assays, classical drop tests were performed. Fresh cells of all tested strains were resuspended in water and adjusted to an optical density [OD_600_] of 1.0. A series of 10-fold dilutions were prepared from each suspension, and 3 μl aliquots were spotted on agar plates as described [59].

### Bacterial strains and vector cloning

*Escherichia coli* strain Top10F’ (Invitrogen, Groningen, The Netherlands) was used for plasmid propagation. PCR products of *FfNPR1-1*, *FfNPR1-3* and *FfGAP1* were cloned into the vector pCR2.1-TOPO using the TOPO TA Cloning Kit (Invitrogen, Groningen, The Netherlands). For *FfNPR1-1* gene replacement, a 0.6 kb fragment from the 5´-region was amplified with primers NPR-LFF / NPR-LFR and a 0.715 kb fragment from the 3´-non-coding region was amplified with primers npr-RFF and npr-RFR, respectively. The SacI/XbaI-digested 5’ flank and the SalI/XhoI- digested 3’ flank were cloned into the plasmid pNR1 carrying a nourseothricin resistance cassette [76]. The *FfGAP1* deletion vector pΔFfGAP1 was constructed by cloning a 0.7 kb SacI/NotI fragment amplified with primers FfGAP1-GR1-SacI and FfGAP1-GR2-NotI into the SacI/NotI digested pNR1 vector [76], and in a second step a 1.0 kb HindIII/XhoI fragment amplified with primers FfGAP1-GR3-Hind and FfGAP1-GR4-Xho into the HindIII/XhoI restricted vector. The replacement vectors pΔNPR1-2 and pΔNPR1-3 were generated via the yeast homologous recombination system [74,75]. To create pΔNPR1-2, the flanking regions of *FfNPR1-2* were amplified using the primer pair NPR1-2-KO-LF-for/NPR1-2-KO-LF-rev for the upstream region and NPR1-2-KO-RF-for/NPR1-2-KO-RF-rev for the downstream region of *FfNPR1-2*, while the hygromycin resistance cassette was amplified from pCSN44 [77] using the primer pair hphF/hphR. All three fragments were cloned into the EcoRI/XhoI-restricted pRS426 [78]. For transformation, the replacement fragment was amplified from vector pΔNPR1-2 with the primer pair NPR1-2-KO-LF-for and NPR1-2-KO-RF-rev.

To create pΔNPR1-3, the flanking regions of *FfNPR1-3* were amplified using the primer pair NPR1-3-KO-LF-for/NPR1-3-KO-LF-rev for the upstream region and NPR1-3-KO-RF-for/NPR1-3-KO-RF-rev for the downstream region of *FfNPR1-3*, while the geneticin resistance cassette was amplified from pKS-Gen [79] using the primer pair Geni-gpd-F and Geni-tubT-R. All three fragments were cloned into the EcoRI/XhoI-restricted vector pRS426 [78]. For transformation, the replacement fragment was amplified from vector pΔNPR1-3 by using the primer pair NPR1-3-KO-LF-for/NPR1-3-KO-RF-rev.

For complementing the *S*. *cerevisiae* mutant strain M4276 with *GAP1* homologous genes from *F*. *fujikuroi*, full length cDNA fragments of the AAP-encoding genes *FFUJ_09118*, *FFUJ_11370*, *FFUJ_05331* and *FFUJ_01136* were amplified with the primers FFUJ_09118-PstI/FFUJ_09118-Not, FFUJ_11370-Sal/FFUJ_11370-Not, FFUJ_05331-Sal/FFUJ_05331-Not, and FFUJ_01136-Pst/FFUJ_01136-Not, respectively, and cloned into the accordingly restricted vector yEX-C [39]. The empty vector was used as a negative control in transformations of *S*. *cerevisiae* M4276, whereas yEXPcGAP1 [39] was used as a positive control.

pAS103 [33] containing the *S*. *cerevisiae* HA-tagged *NPR1* gene under the control of the endogenous promoter was used as a positive control for complementation of the *S*. *cerevisiae NPR1* mutant 30788a. For cloning the *F*. *fujikuroi NPR1* homologous genes behind the yeast NPR1 promoter, vector pHA-NPR1 [33] carrying the yeast *NPR1* gene with its native promoter, but without the *NPR1* terminator, was used as basis vector. To remove the yeast *NPR1* gene, the vector was digested with XbaI and religated, resulting in plasmid YEplacRL-Xba. The *NPR1* terminator was amplified from *S*. *cerevisiae* genomic DNA with primers YNPR1-Term-Xba-F/YNPR1-Term-Pst1-R and cloned into the XbaI/PstI-digested vector YEplacRL-Xba. The resulting YEplac195-npr1-Term was restricted with XbaI and used for cloning the *F*. *fujikuroi NPR1* homologous genes by yeast recombination cloning. For this cloning approach, the full length fragments of *FfNPR1-1*, *FfNPR1-2* or *FfNPR1-3* were amplified with the primer pairs NPR1Y-for/NPR1Y-rev, NPR2Y-for/NPR2Y-rev or NPR3Y-for/NPR3Y-rev, respectively, and cloned between the yeast *ScNPR1* promotor and the yeast *ScNPR1* terminator into the vector YEplac195-NPR1-Term.

For generating the pFfGAP1-GFP fusion vectors, a full-length cDNA clone of *FfGAP1* was amplified using the primers FfGAP1-GFP-F-WT and FfGAP1-GFP-R that contain overlapping sequences homologous to the vector pNAN-OGG [75]. This vector contains a hygromycin resistance cassette, and a codon-optimized e*GFP* [77,80] under control of the *Aspergillus nidulans oliC* promoter and the *gluc* terminator. The *FfGAP1* PCR product and the NcoI-digested plasmid pNAH-OGG were co-transformed into *S*. *cerevisiae* yielding pFfGAP1-GFP. The point-mutated *FfGAP1* gene copy (lysine 7 and lysine 15 were exchanged for alanine) was generated by the same approach, but by using primer FfGAP1-GFP-F-Mut in combination with FfGAP1-GFP-R for amplification of the mutated gene allele. DNA of pooled yeast colonies was isolated as described above and transformed into *E*. *coli*. Plasmid DNA from single colonies was isolated and sequenced.

Both vectors were transformed into the Δ*FfGAP1* mutant. Integration of the whole FfGAP1-GFP fusion constructs was controlled by diagnostic PCR.

### Nucleic acid isolation and analysis

Lyophilized mycelium was ground into a fine powder and dispersed (in the case of DNA for use in PCR) in extraction buffer as described by Cenis [81]. DNA for Southern hybridization experiments was prepared following the protocol of Doyle and Doyle [82]. Plasmid DNA was extracted using the Genomed plasmid extraction kit (Genomed, Löhne, Germany). Total *F*. *fujikuroi* RNA was isolated using the RNAgents total RNA isolation kit (Promega, Mannheim, Germany). For Southern analysis, genomic DNA was digested to completion with appropriate restriction enzymes, fractionated in 1.0% (w/v) agarose gels, and transferred to nylon membranes (N+, Amersham). DNA probes were randomly labelled and hybridizations were carried out overnight at 65°C.

### PCR and RT-PCR

PCR reactions contained 25 ng DNA, 5 pmol of each primer, 200 μM desoxynucleotide triphosphates, 1 unit BioTherm DNA polymerase and 1x concentration of BioTherm buffer (Genecraft GmbH, Lüdinghausen, Germany). The reactions were started with 4 min at 94°C, followed by 35 cycles of 1 min per kb of the product at 94°C, 1 min at 56° to 65°C, 1 min at 70°C, and a final 10 min at 70°C. PCR products were cloned into pCR2.1-TOPO (Invitrogen). Resistance cassettes and *eGFP* for yeast recombination were amplified with the proofreading Phusion DNA polymerase (Finnzymes, Vantaa, Finland). These reactions contained 25 ng DNA, 5 pmol of each primer, 200 μM desoxynucleotide triphosphates, 1 unit Phusion polymerase and 1x concentration of HF-buffer. The reactions were started with 5 min at 95°C, followed by 35 cycles of 1 min at 94°C, 1 min at 56° to 65°C, 1 min per kb of the product at 72°C, and a final 10 min at 72°C. Primers are listed in S2 Table.

### Site-directed mutagenesis

Site-directed mutagenesis was carried out as described by the manufacturer using the QuikChange II Site-Directed Mutagenesis Kit (Agilent Technologies). To generate an *FfGAP1* template vector for site directed mutagenesis, the full length *FfGAP1* cDNA fragment from *F*. *fujikuroi* was amplified from cDNA by using the RT-primers FfGAP1-WT-F1 and FfGAP1-WT-R1 (S2 Table), and the PCR fragment was cloned into vector pCR2.1-TOPO (Invitrogen) resulting in vector pGAP1-cDNA. Primers for introducing specific point mutations into the wild-type *FfGAP1* cDNA sequence are listed in S1 Table. The mutated gene copy was then cloned into the destination vector containing the *eGFP* gene and subsequently transformed into the wild-type and Δ*NPR1* mutants.

### Fungal transformations

Preparation of protoplasts of *F*. *fujikuroi* was carried out as described [83]. For deletion of *FfGAP1* and single or double deletion of *NPR1-*homologous genes, strain IMI58289 or single *NPR1* deletion strains were transformed with 10 μg of the *FfGAP1* or the *FfNPR1-1*, *FfNPR1-2* and *FfNPR1-3* replacement fragments of the pΔGAP1 or the pΔNPR1 replacement vectors, respectively. Transformed protoplasts were regenerated at 28°C in a complete regeneration agar (0.7 M sucrose, 0.5 g/l yeast extract) with 100 μg/ml nourseothricin (Werner Agents, Jena, Germany), 100 μg/ml hygromycin or 100 μg/ml geneticin (Sigma-Aldrich, Taufkirchen, Germany) for 6 to 7 days as specified above. Single spore cultures for purification of the heterokaryons were established from the transformants with homologous integration of the replacement cassettes and used for DNA isolation and subsequent PCR and Southern blot analysis.

For FfGap1 localization studies, 107 protoplasts of strain IMI58289, Δ*FfNPR1-1* or Δ*FfNPR1-3* were transformed with 10 μg of vector pGAP1-Gfp carrying the *FfGAP1-GFP* construct or pGAP1K7/15A-GFP carrying the mutated *FfGAP1K7/K15-GFP* construct. The transformed protoplasts were regenerated and selected as described above. For yeast transformation, yeast cells were treated with lithium acetate [84] and transformed according to the method of Sherman et al. [85].

### Western blot analysis

Membrane-enriched cell extracts of yeast were prepared as previously described [18]. For blot analysis, equal protein amounts were loaded onto an 8% SDS-polyacrylamide gel in a Tricine system [86].

For detection of Gap1, Mep2 and Pma1, polyclonal anti-Gap1, anti-Mep2 and anti-Pma1 antibodies raised in rabbits were used at 1:5000 dilution [27, 87]. To detect the primary antibodies HRP (Horse radish peroxidase) conjugated anti-rabbit secondary antibodies were used in a 1:10000 dilution, followed by visualization of the occurring chemoluminescence.

### *In silico* identification of potential AAPs

The putative AAP proteins were identified in the genome database of the *F*. *fujikuroi* wild-type strain IMI58289 [7] by a BlastP search [35] using the protein sequences of *S*. *cerevisiae* Gap1 and by searching for following InterPro domains: IPR004841 (amino acid permease domain); IPR004840 (conserved amino acid permease site); IPR004762 (fungal amino acid permease); and IPR013057 (transmembrane amino acid transporter).

### Fluorescence microscopy

10 μl of *Fusarium fujikuroi* mycelium, grown in liquid ICI media (with nitrogen source dependent on the experiment), was directly used for microscopy. For staining of vacuolar membranes the endocytosis marker FM 4–64 (Life Technologies, Germany) was used. 10 μl of a 10 μg/ml stock solution (in ICI medium) were added to the cells and incubated for 15–20 min for staining of vacuolar membranes [52,53,88]. GFP-fluorescence and FM 4–64 staining were observed using a Leica DMRBE microscope (Leica, Wetzlar, Germany) equipped with a high-performance charge-coupled device 12 bit SensiCam (PCO AG, Kehlheim, Germany) and filter set L5 (excitation band-pass filter 480/40, dichromatin mirror 505, suppression band-pass filter 527/30) for GFP or filter set 43 HE (excitation band-pass filter 550/25, beam splitter FT 570, emission band-pass filter 605/70) for FM 4–64, respectively.

## Supporting Information

S1 Fig Amino acid sequences of three putative *F*. *fujiku*roi Npr1-like proteins and *S*. *cerevisiae* Npr1. The presence of multiple serine residues is highlighted in red.
(TIF)

S2 Fig Analysis of Δ*FfNPR1-1*, Δ*FfNPR1-2* and Δ*FfNPR1-3* deletion mutants. (A) Southern blot analysis of XbaI-digested genomic DNA of *F*. *fujikuroi* wild-type (Wt), and Δ*FfNPR1-1* transformants T1, T2 and T3. Upon replacement of *FfNPR1-1* by a nourseothricin resistance cassette, a 0.9 kb DNA fragment is hybridized with a radioactive labelled fragment of the 5’ flanking region, instead of a 2.2 kb fragment in case of the wild-type. (B) Southern blot analysis of SacI-digested genomic DNA of *F*. *fujikuroi* wild-type (WT), and Δ*FfNPR1-3* transformants T1 and T2. Upon replacement of *FfNPR1-3* by a geneticin resistance cassette, a 2.8 kb DNA fragment is hybridized with a radioactive labelled fragment of the 5’ flanking region, instead of two fragments of 2.2 kb and 1.6 kb in case of the wild-type. (C) Diagnostic PCR of *F*. *fujikuroi* wild-type (WT) and Δ*FfNPR1-2* transformants T1 and T2. Homologous integration of the *FfNPR1-2* deletion construct was confirmed by diagnostic PCR with primers pCSN44-trpC-P / ΔNPR1-2-KO-dia-rev for the right flank (RF) and primers pCSN44-hph-trpC-T / ΔNPR1-2-KO-dia-for for the left flank (LF). Complete substitution of the wild-type gene was confirmed with primers NPR1-2-WT-for / NPR1-2-WT-rev (WT).
(TIF)

S3 Fig Growth assay of Δ*FfNPR1-3* complementation mutant (Δ*FfNPR1-3* ^*C*^ *)* on different nitrogen sources. Strains were grown on solid ICI minimal medium with either no nitrogen (-N) or the indicated concentrations of various nitrogen sources at 28°C for 4 days.
(TIF)

S4 Fig Impact of nitrogen quality on the speed of intracellular FfGap1-sorting. *F*. *fujikuroi* wild-type (A), Δ*NPR1-1* (B) and Δ*NPR1-3* (C) transformed with a FfGap1-GFP fusion construct were cultivated in liquid ICI medium with 6 mM glutamine for 48 h. Cells were observed by fluorescence (GFP) and brightfield microscopy (BF) before (- N) and up to 4.5 h after addition of 12 mM ammonium sulfate (NH_4_) or 12 mM sodium nitrate (NO_3_).
(TIF)

S1 Table List of putative amino acid permeases in the genome of *F*. *fujikuroi*. (DOCX)

S2 Table Primer used in this study. (DOCX)
