# Supplementary figures and images for: The General Amino Acid Permease FfGap1 of Fusarium fujikuroi Is Sorted to the Vacuole in a Nitrogen-Dependent, but Npr1 Kinase-Independent Manner
Source: PLoS One. 2015 Apr 24;10(4):e0125487. doi: 10.1371/journal.pone.0125487 (PMC4409335; doi:10.1371/journal.pone.0125487)

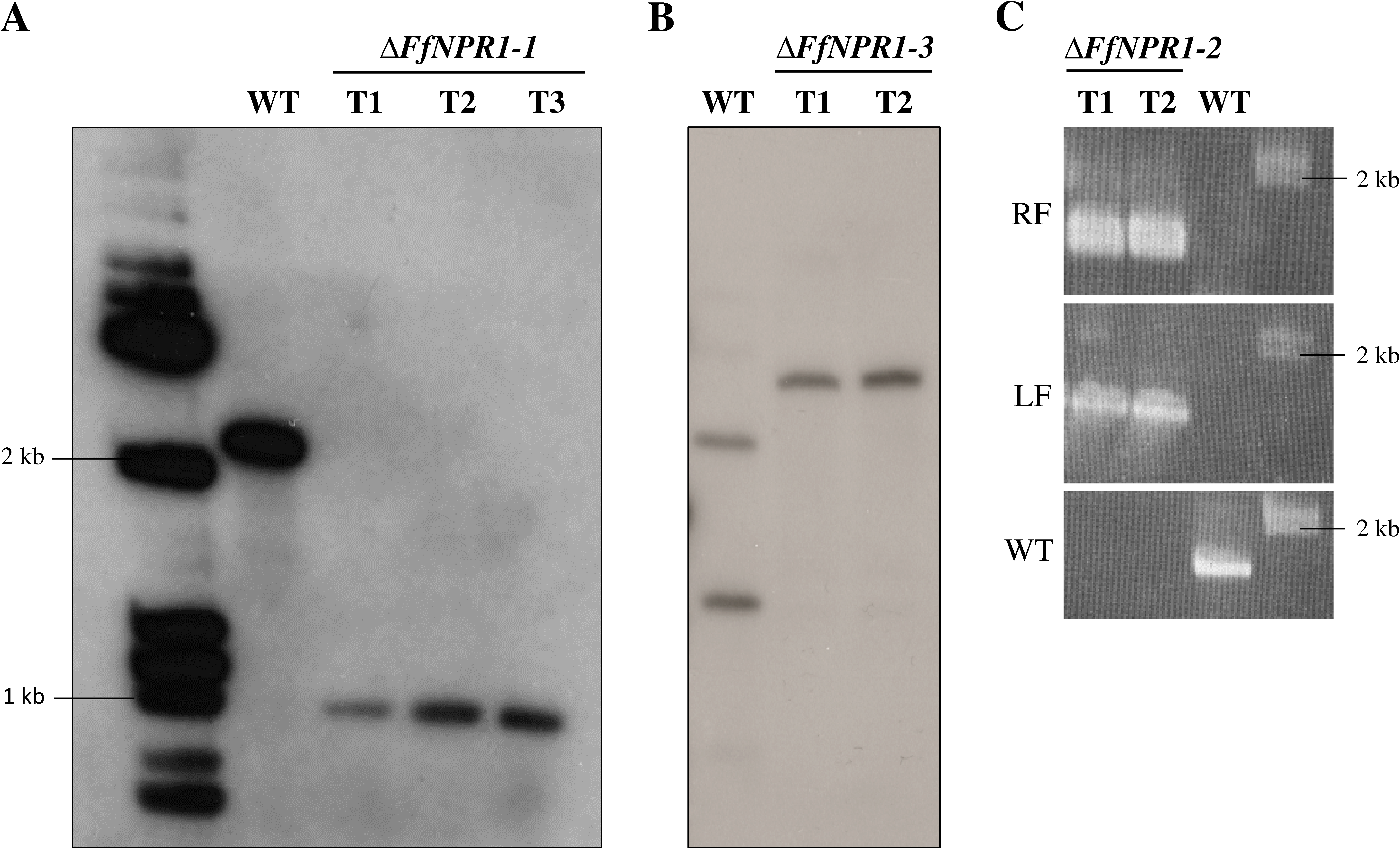

Supplement: S2 Fig — (A) Southern blot analysis of XbaI-digested genomic DNA of F. fujikuroi wild-type (Wt), and ΔFfNPR1-1 transformants T1, T2 and T3. Upon replacement of FfNPR1-1 by a nourseothricin resistance cassette, a 0.9 kb DNA fragment is hybridized with a radioactive labelled fragment of the 5’ flanking region, instead of a 2.2 kb fragment in case of the wild-type. (B) Southern blot analysis of SacI-digested genomic DNA of F. fujikuroi wild-type (WT), and ΔFfNPR1-3 transformants T1 and T2. Upon replacement of FfNPR1-3 by a geneticin resistance cassette, a 2.8 kb DNA fragment is hybridized with a radioactive labelled fragment of the 5’ flanking region, instead of two fragments of 2.2 kb and 1.6 kb in case of the wild-type. (C) Diagnostic PCR of F. fujikuroi wild-type (WT) and ΔFfNPR1-2 transformants T1 and T2. Homologous integration of the FfNPR1-2 deletion construct was confirmed by diagnostic PCR with primers pCSN44-trpC-P / ΔNPR1-2-KO-dia-rev for the right flank (RF) and primers pCSN44-hph-trpC-T / ΔNPR1-2-KO-dia-for for the left flank (LF). Complete substitution of the wild-type gene was confirmed with primers NPR1-2-WT-for / NPR1-2-WT-rev (WT). (TIF) [file pone.0125487.s002.tif]

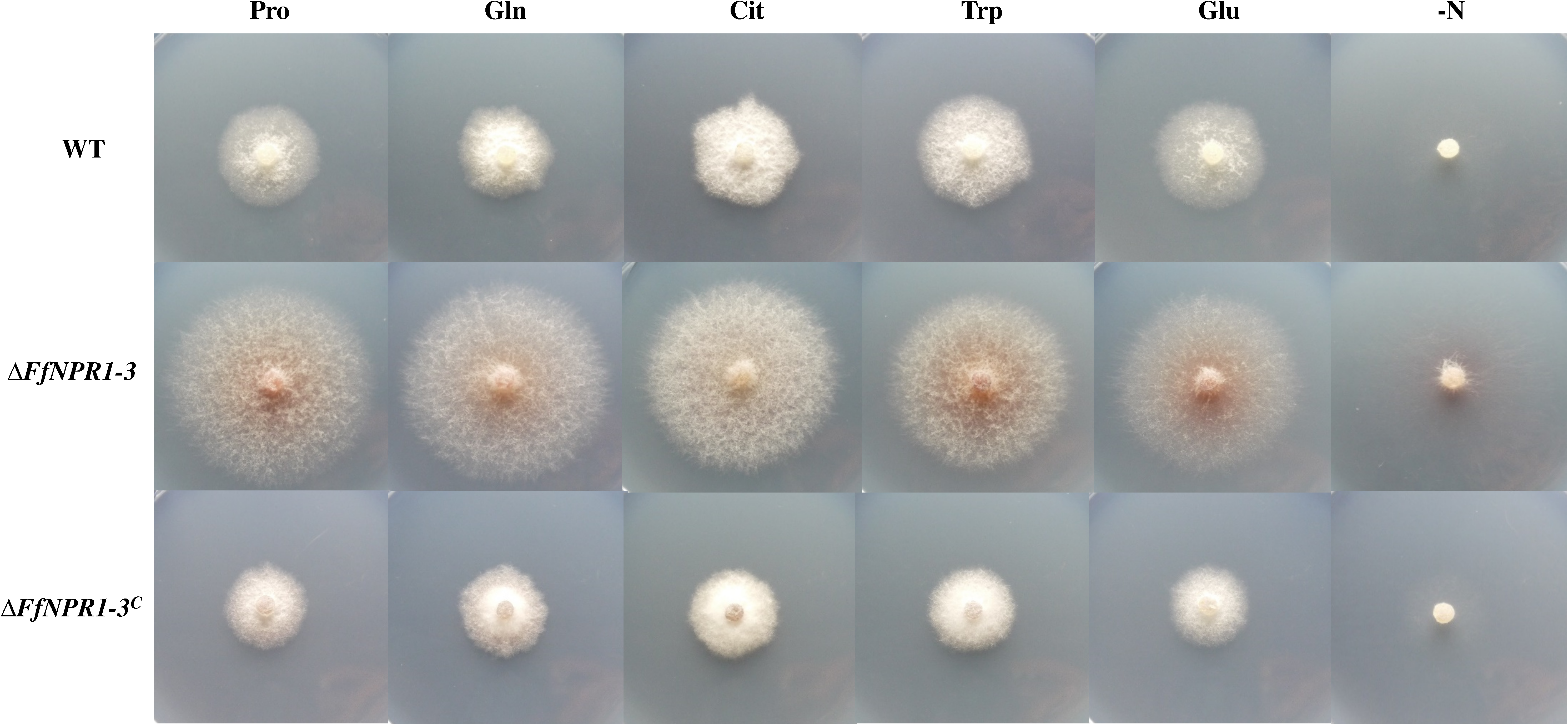

Supplement: S3 Fig — Strains were grown on solid ICI minimal medium with either no nitrogen (-N) or the indicated concentrations of various nitrogen sources at 28°C for 4 days. (TIF) [file pone.0125487.s003.tif]

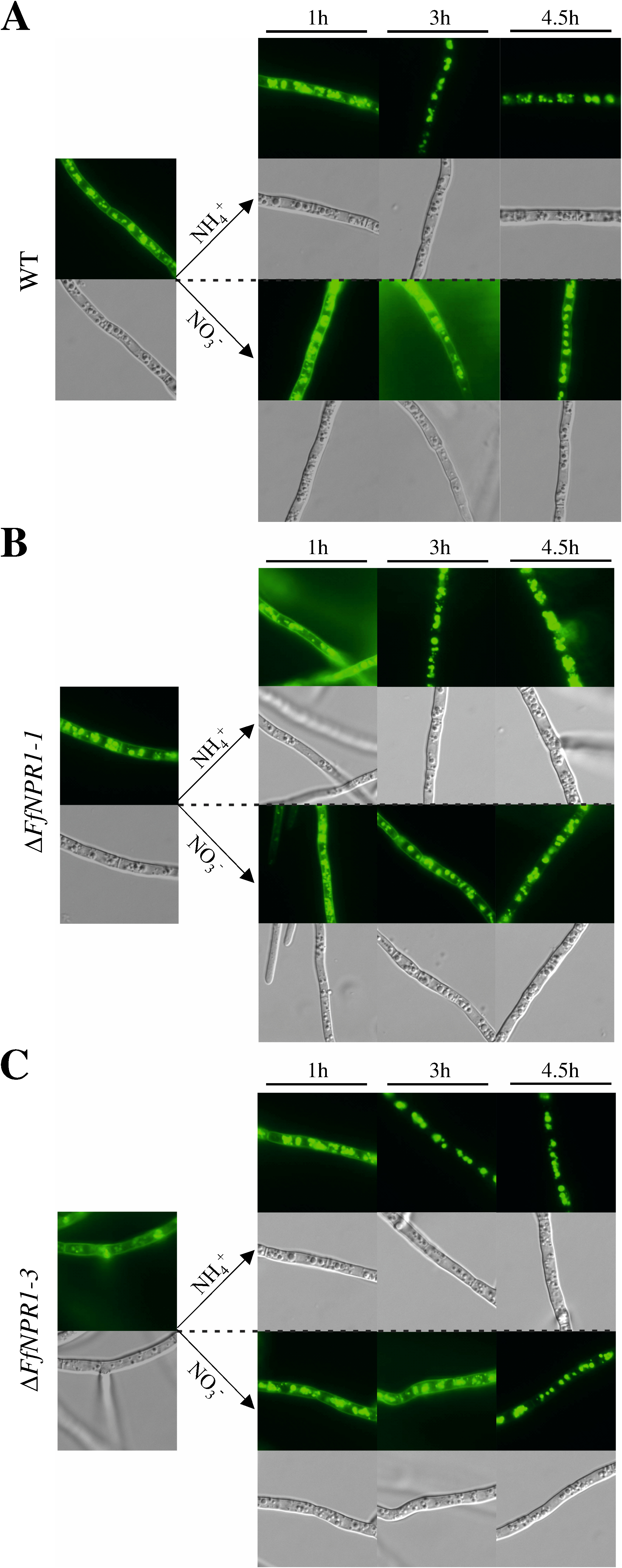

Supplement: S4 Fig — F. fujikuroi wild-type (A), ΔNPR1-1 (B) and ΔNPR1-3 (C) transformed with a FfGap1-GFP fusion construct were cultivated in liquid ICI medium with 6 mM glutamine for 48 h. Cells were observed by fluorescence (GFP) and brightfield microscopy (BF) before (- N) and up to 4.5 h after addition of 12 mM ammonium sulfate (NH4) or 12 mM sodium nitrate (NO3). (TIF) [file pone.0125487.s004.tif]
