# Supplementary material for: The General Amino Acid Permease FfGap1 of Fusarium fujikuroi Is Sorted to the Vacuole in a Nitrogen-Dependent, but Npr1 Kinase-Independent Manner
Source: PLoS One. 2015 Apr 24;10(4):e0125487. doi: 10.1371/journal.pone.0125487 (PMC4409335; doi:10.1371/journal.pone.0125487)
Supplement: S1 Table — (DOCX) [file pone.0125487.s005.docx]

| ***Gene Code*** | ***Putative GAP*** | ***Gene Description*** | ***AA Length*** | ***Interpro ID*** |
| --- | --- | --- | --- | --- |
| FFUJ_00045 |  | choline permease | 511 | IPR002293; IPR004840 |
| FFUJ_00095 |  | related to HNM1 - Choline permease | 514 | IPR002293 |
| FFUJ_00190 |  | related to neutral amino acid permease | 483 | IPR013057 |
| FFUJ_00265 |  | related to HNM1 - Choline permease | 527 | IPR002293 |
| FFUJ_00914 |  | related to UGA4 - GABA permease - also involved in delta-aminolevulinate transport | 514 | IPR002293 |
| FFUJ_01137 | Putative GAP | probable amino acid transport protein GAP1 | 604 | IPR002293; IPR004762; IPR004840; IPR004841 |
| FFUJ_01585 |  | probable AVT3 - involved in amino acid efflux from the vacuole | 598 | IPR013057 |
| FFUJ_01902 | Putative GAP | probable DIP5 - glutamate and aspartate permease | 578 | IPR002293; IPR004840; IPR004841 |
| FFUJ_02047 |  | related to HNM1 - Choline permease | 536 | IPR002293; IPR004840 |
| FFUJ_02178 |  | related to amino acid permease | 641 | IPR002293; IPR004841 |
| FFUJ_02249 |  | related to neutral amino acid permease | 475 | IPR013057 |
| FFUJ_02336 |  | related to large neutral amino acid transporter | 514 | IPR002293 |
| FFUJ_02389 |  | related to high affinity methionine permease | 537 | IPR002293 |
| FFUJ_02419 |  | related to high affinity methionine permease | 688 | IPR002293 |
| FFUJ_03330 |  | related to neutral amino acid permease | 509 | IPR013057 |
| FFUJ_03458 |  | related to GABA permease | 521 | IPR002293 |
| FFUJ_03472 |  | probable neutral amino acid permease | 478 | IPR013057 |
| FFUJ_03507 | Putative GAP | probable lysine permease | 516 | IPR002293; IPR004840; IPR004841 |
| FFUJ_03523 |  | related to GABA permease | 526 | IPR002293; IPR004840 |
| FFUJ_03687 |  | related to HNM1 - Choline permease | 533 | IPR002293 |
| FFUJ_03718 |  | HNM1 - Choline permease | 513 | IPR002293 |
| FFUJ_03747 |  | related to HNM1 - Choline permease | 523 | IPR002293 |
| FFUJ_03818 |  | related to HNM1 - Choline permease | 514 | IPR002293 |
| FFUJ_04023 |  | probable neutral amino acid permease | 471 | IPR013057 |
| FFUJ_04069 |  | probable carnitine transporter | 565 | IPR002293; IPR004841 |
| FFUJ_04110 |  | probable general amino acid permease | 559 | IPR002293; IPR004841 |
| FFUJ_04287 |  | probable AVT6 - involved in amino acid efflux from the vacuole | 507 | IPR013057 |
| FFUJ_05331 | Putative GAP | probable GAP1 - General amino acid permease | 562 | IPR002293; IPR004840; IPR004841 |
| FFUJ_05835 |  | choline permease | 532 | IPR002293; IPR004840 |
| FFUJ_06434 |  | related to GABA transport protein | 564 | IPR002293 |
| FFUJ_06575 |  | related to neutral amino acid permease | 463 | IPR013057 |
| FFUJ_06576 |  | related to high affinity methionine permease | 589 | IPR002293 |
| FFUJ_06819 |  | related to high affinity methionine permease | 608 | IPR002293 |
| FFUJ_07055 | Putative GAP | related to lysine permease | 529 | IPR002293; IPR004840; IPR004841 |
| FFUJ_07073 |  | related to neutral amino acid permease | 516 | IPR013057 |
| FFUJ_07634 | Putative GAP | probable amino acid transporter | 546 | IPR002293; IPR004840; IPR004841 |
| FFUJ_07760 |  | related to GABA permease | 529 | IPR002293 |
| FFUJ_07889 |  | related to amino acid transport protein | 679 | IPR013057 |
| FFUJ_08080 | Putative GAP | related to general amino acid permease | 556 | IPR002293; IPR004841 |
| FFUJ_08106 |  | probable general amino acid permease | 563 | IPR002293; IPR004841 |
| FFUJ_08308 |  | related to amino acid transport protein GAP1 | 562 | IPR002293; IPR004841 |
| FFUJ_08309 | Putative GAP | NAAP-1 amino acid permease NAAP1 | 576 | IPR002293; IPR004840; IPR004841 |
| FFUJ_08616 |  | probable proline-specific permease (proline transport protein) | 405 | IPR002293; IPR004841 |
| FFUJ_08705 | Putative GAP | probable GAP1 - General amino acid permease | 537 | IPR002293; IPR004840; IPR004841 |
| FFUJ_08913 | Putative GAP | probable amino acid permease NAAP1 | 578 | IPR002293; IPR004840; IPR004841 |
| FFUJ_09058 |  | related to neutral amino acid permease | 480 | IPR013057 |
| FFUJ_09097 |  | probable neutral amino acid permease | 470 | IPR013057 |
| FFUJ_09118 | Putative GAP | probable GAP1 - General amino acid permease | 541 | IPR002293; IPR004762; IPR004840; IPR004841 |
| FFUJ_09356 | Putative GAP | probable amino acid transport protein GAP1 | 583 | IPR002293; IPR004762; IPR004840; IPR004841 |
| FFUJ_09391 |  | probable carnitine transporter | 541 | IPR002293; IPR004840; IPR004841 |
| FFUJ_09486 | Putative GAP | related to general amino acid permease | 529 | IPR002293; IPR004841 |
| FFUJ_09628 |  | probable carnitine transporter | 566 | IPR002293; IPR004841 |
| FFUJ_09866 |  | related to YBL089w | 549 | IPR013057 |
| FFUJ_09952 |  | related to neutral amino acid permease | 489 | IPR013057 |
| FFUJ_10091 | Putative GAP | probable amino acid permease NAAP1 | 582 | IPR002293; IPR004840; IPR004841 |
| FFUJ_10307 |  | choline permease | 529 | IPR002293 |
| FFUJ_10331 |  | related to neutral amino acid permease | 474 | IPR013057 |
| FFUJ_10350 |  | related to HNM1 - Choline permease | 519 | IPR002293 |
| FFUJ_10530 |  | related to neutral amino acid permease | 471 | IPR013057 |
| FFUJ_10811 |  | related to HNM1 - Choline permease | 507 | IPR002293 |
| FFUJ_10830 |  | probable neutral amino acid permease | 460 | IPR013057 |
| FFUJ_10948 |  | probable general amino acid permease | 577 | IPR002293; IPR004841 |
| FFUJ_10985 |  | related to high affinity methionine permease | 597 | IPR002293 |
| FFUJ_11132 |  | probable general amino acid permease | 564 | IPR002293; IPR004841 |
| FFUJ_11142 |  | probable general amino acid permease | 529 | IPR002293; IPR004841 |
| FFUJ_11370 | Putative GAP | probable amino acid transport protein GAP1 | 588 | IPR002293; IPR004762; IPR004840; IPR004841 |
| FFUJ_11387 | Putative GAP | probable amino acid transport protein GAP1 | 620 | IPR002293; IPR004762; IPR004840; IPR004841 |
| FFUJ_11468 |  | related to neutral amino acid permease | 466 | IPR013057 |
| FFUJ_11595 |  | related to high affinity methionine permease | 584 | IPR002293 |
| FFUJ_11624 | Putative GAP | probable amino acid permease NAAP1 | 572 | IPR002293; IPR004841 |
| FFUJ_11640 | Putative GAP | probable lysine permease | 518 | IPR002293; IPR004840; IPR004841 |
| FFUJ_11723 |  | related to HNM1 - Choline permease | 521 | IPR002293 |
| FFUJ_11749 |  | related to neutral amino acid permease | 488 | IPR013057 |
| FFUJ_11817 |  | related to neutral amino acid permease | 466 | IPR013057 |
| FFUJ_11839 |  | related to GABA permease | 522 | IPR002293 |
| FFUJ_12124 |  | related to HNM1 - Choline permease | 516 | IPR002293 |
| FFUJ_12193 |  | related to neutral amino acid permease | 478 | IPR013057 |
| FFUJ_12515 |  | choline permease | 530 | IPR002293 |
| FFUJ_12528 |  | related to HNM1 - Choline permease | 1045 | IPR002293 |
| FFUJ_12724 |  | related to amino acid permease 2 (AAP-2) | 541 | IPR002293 |
| FFUJ_13022 |  | related to HNM1 - Choline permease | 530 | IPR002293; IPR004840 |
| FFUJ_13323 |  | related to large neutral amino acid transporter | 611 | IPR002293 |
| FFUJ_13381 |  | related to na+/k+/2cl- cotransporter | 1340 | IPR004841 |
| FFUJ_13382 |  | probable AVT3 - involved in amino acid efflux from the vacuole | 767 | IPR013057 |
| FFUJ_13762 |  | related to neutral amino acid permease | 508 | IPR013057 |
| FFUJ_13886 |  | probable MUP1 - High affinity methionine permease | 523 | IPR002293 |
| FFUJ_13909 |  | probable high affinity methionine permease | 531 | IPR002293 |
| FFUJ_13970 |  | related to cholin permease | 512 | IPR002293 |
| FFUJ_13982 |  | related to GABA transport protein | 532 | IPR002293 |
| FFUJ_13995 |  | probable neutral amino acid permease | 470 | IPR013057 |
| FFUJ_14280 |  | related to neutral amino acid permease | 466 | IPR013057 |
| FFUJ_14306 |  | related to HNM1 - Choline permease | 519 | IPR002293 |
| FFUJ_14413 |  | related to neutral amino acid permease | 476 | IPR013057 |
| FFUJ_14571 |  | probable carnitine transport protein | 563 | IPR002293; IPR004841 |
| FFUJ_14583 |  | related to cholin permease | 523 | IPR002293 |
| FFUJ_14649 |  | related to HNM1 - choline permease | 556 | IPR002293 |
| FFUJ_14656 |  | related to cholin permease | 528 | IPR002293; IPR004840 |
| FFUJ_14831 |  | related to neutral amino acid permease | 471 | IPR013057 |
| FFUJ_14854 | Putative GAP | probable general amino acid permease | 537 | IPR002293; IPR004841 |
