# Supplementary material for: The General Amino Acid Permease FfGap1 of Fusarium fujikuroi Is Sorted to the Vacuole in a Nitrogen-Dependent, but Npr1 Kinase-Independent Manner
Source: PLoS One. 2015 Apr 24;10(4):e0125487. doi: 10.1371/journal.pone.0125487 (PMC4409335; doi:10.1371/journal.pone.0125487)
Supplement: S2 Table — (DOCX) [file pone.0125487.s006.docx]

| **primer name** | **Sequence** **(5‘ 3‘)** |
| --- | --- |
| FfGAP1-GR1-SacI | ATGAGCTCGTGCCCTATGTTTGCTTGGCAGGG |
| FfGAP1-GR2-NotI | ATAGCGGCCGCATGCTCTGCAGTGTTGTTACTGTGGC |
| FfGAP1-GR3-Hind | ATAAAGCTTAAGTTGTATGTCAAGGCCATTGATATGG |
| FfGAP1-seq1-for | GCTTATCTTAAGCTTGTTCG |
| FfGAP1-seq2-for | CGTCACTTCAGGATCAATGC |
| FfGAP1-seq3-for | GTCGAATTCGTCCTGTCCG |
| FfGAP1-seq4-for | GATGCAGGCATCAAGGTTCTG |
| FfGAP1-WT-F1 | GGTGCTTTCACCAACTTCAAGGG |
| FfGAP1-WT-R1 | AAAGACTCAGCATCGGGAGGAGAGCC |
| FfGAP1-GFP-F-Mut | CCATCACATCACAATCGATCCAACCATGTCGTCGTCACCGCACGCGGATGAGGTGAATTACGAGACCGCGGACGAGACCACGGTCACGTCGCC |
| FfGAP1-GFP-F-WT | CCATCACATCACAATCGATCCAACCATGTCGTCGTCACCGCACAAGGATGAGGTGAATTACGAGACCAAGGACGAGACCACGGTCACGTCGCC |
| FfGAP1-GFP-R | TACTTACCTCACCCTTGGAAACCATGATAATAGCCGAGAGCAGACGTCTTGGCGCCG |
| FfGAP1-GR4-XhoI | AATCTCGAGGCCCGAAAACATTCCCTGGTTGTAACCG |
| FFUJ_01136-RT1-PstI | GCTGCAGGAATGGCGCCTCACGATGT |
| FFUJ_01136-RT2-NotI | ATGCGGCCGCTCAGCACATGAACTTGTAATATC |
| FFUJ_05331-cDNA-for2 | CCGTCGACCCTCCTCCTGCCTCGTCACGATGAGTC |
| FFUJ_05331-cDNA-rev | GCTAAGAAACTCCTTACTGCGGCCGCCTG |
| FFUJ_09118-RT1-PstI | CACTGCAGAGCATGTCGTCGTCACCC |
| FFUJ_09118-RT2-NotI | TCTGCGGCCGCTATCTAGATAATAGCCGAGCAGACGTCTTGG |
| FFUJ_11370-RT1-Sal | ATGTCGACCATGGCATACCAACGCGAGTTCGAGATGC |
| FFUJ_11370-RT2-NotI | GTAGCGGCCGCAGTTCACAACTGCAATTTGTATGCATGG |
| Geni-gpd-F | GTCGGAGACAGAAGATGATATTGAAGGAGCCAACAAAACACAGTTCCGACCAC |
| Geni-tubT-R | GTTGGAGATTTCAGTAACGTTAAGTGGATATCATCATGCAACATGCATGTACTG |
| GFP-yeast-for | AGCTCTGTACAGTGACCGGTGACAAGCTTCGCTCCATCGCCACGCGCTCCATGGTGAGCAAGGGCGAGGAGCT |
| GFP-yeast-rev | GCGGATAACAATTTCACACAGGAAACAGCGCATGCCTGCAGGTCGAGTGGAG |
| hphF | GTCGGAGACAGAAGATGATATTGAAGGAGC |
| hph-OE-Prom | GTCACCGGTCACTGTACAGAGCTGACAGAAGATGATATTGAAGGAGC |
| hph-OE-Term | GTAACGCCAGGGTTTTCCCAGTCACGACGGATTTCAGTAACGTTAAGTGGAT |
| hphR | GTTGGAGATTTCAGTAACGTTAAGTGGAT |
| M13/pUC reverse (-46) | GAGCGGATAACAATTTCACACAGG |
| M13/pUC universe (-40) | GTTTTCCCAGTCACGAC |
| NPR1-seq1-for | GTTCACCAAGTCTCTCCACG |
| NPR1-seq1-rev | CGTGGAGAGACTTGGTGAAC |
| NPR1-seq3-for | CAGCTTCTAGAGAATCTAGTC |
| NPR1-seq4-for | AGACACACCTACGAAACCGA |
| NPR1-seq5-for | GAGGTTTACGACTCTAAGGAG |
| NPR1Y-for | GAAAGCTCTACGAAACATTAATGAGTTCTTCAAATCCTCCCCAG |
| NPR1Y-rev | GTGGGGACGCTTATTTATTGTCACTTCTTCTCGGGTTGTTGC |
| NPR1-2-KO-dia-for | CACCTTGAAGCTTTGCAC |
| NPR1-2-KO-dia-rev | GAGACGTGCGTGCATATCG |
| NPR1-2-KO-LF-for | GTAACGCCAGGGTTTTCCCAGTCACGACGTAGTTCGGTTTGGCTCTGG |
| NPR1-2-KO-LF-rev | ATCCACTTAACGTTACTGAAATCTCCAACGTCCGGTGCTGAGAAATAG |
| NPR1-2-KO-RF-for | CTCCTTCAATATCATCTTCTGTCTCCGACGACTGATGGAACGACATTTG |
| NPR1-2-KO-RF-rev | GCGGATAACAATTTCACACAGGAAACAGCGATGCTTTCCGTCGCTTCTG |
| NPR1-2-RT2-for | GTGCATCGACTGGTCTGACT |
| NPR1-2-seq1-for | GAACCATGTCATGGAATATTG |
| NPR1-2-WT-for | CCAAGAGCCATGGTAGCACC |
| NPR1-2-WT-rev | GGAGTACTTGGTTCGTAGGT |
| NPR1-2Y-for | GAAAGCTCTACGAAACATTAATGGCGGCACTCTCGGCCCCA |
| NPR1-2Y-for | GAAAGCTCTACGAAACATTAATGGCGGCACTCTCGGCCCCA |
| NPR1-2Y-rev | GTGGGGACGCTTATTTATTGCTAGTAGCCCGGCTGGCCTG |
| NPR1-2Y-rev | GTGGGGACGCTTATTTATTGCTAGTAGCCCGGCTGGCCTG |
| NPR1-3-KO-dia-for | CCACCATTACTTCTTTTCACC |
| NPR1-3-KO-dia-rev | TAAGCCTACCGAGCGAGAG |
| NPR1-3-KO-LF-for | GTAACGCCAGGGTTTTCCCAGTCACGACGCATCTCCGTCTTGTCCTTGC |
| NPR1-3-KO-LF-rev | ATCCACTTAACGTTACTGAAATCTCCAACCTGCCACTGTCTCTGTCTGTC |
| NPR1-3-KO-RF-for | CTCCTTCAATATCATCTTCTGTCTCCGACGAGCGATTCGAACTCGATGAG |
| NPR1-3KO-RF-rev | GCGGATAACAATTTCACACAGGAAACAGCCAATACGTGAAGTATCGTGC |
| NPR1-3-RT1-for | GCAGATGATGCGAGGAGTCG |
| NPR1-3-RT1-rev | CTCTCCACATATCCGAGAGC |
| NPR1-3-seq1-for | GCTCTCGGATATGTGGAGAG |
| NPR1-3-WT-for | CCACAGAATGGTGCAACTAC |
| NPR1-3-WT-rev | CATAGACCCTCTTCACCAGC |
| NPR1-3Y-for | GAAAGCTCTACGAAACATTAATGCCCACTGCATCTGGTTCCTCA |
| NPR1-3Y-rev | GTGGGGACGCTTATTTATTGTCATAGACCCTCTTCACCAGCCT |
| NPR-LF-check | GAGCGAGACCTAAATCGGCTCAGATCG |
| NPR-LFF-SacI | CATATTCATGGAGCTCGGACTGTTTGCGTG |
| NPR-LFR-XbaI | ATGCGGGTTCTAGAAACACGCTAGATGTG |
| NPR-RF-check | CTAAGTCTCCTCATGGCTCCCAATTGCC |
| NPR-RFF-SalI | TGACAACCACGTCGACAACTTGGTAAAGC |
| NPR-RFR-XhoI | AAGCTAGATACTCGAGACCGAAGCATTGG |
| pCSN44-hph-trpC-T | GGAATAGAGTAGATGCCGACCGG |
| pCSN44-trpC-P | CCTCCACTAGCTCCAGCCAAGCCC |
| pKS-Gen-gpd-P | GGTGATGAGCAGGTGGTGAGAGG |
| pKS-Gen-tub-T | CCTGTCAGACACTCTAGTTGTTGAC |
| pLOF-OliP | GGTACTGCCCCACTTAGTGGCAGCTCGCG |
| Tub-T2 | GGTCCTCGGAGTGCAGATGGG |
| YNPR1-Term-dia-rev | CAGGAGGTCAATCTATTTAGGC |
| YNPR1-Term-Pst1-rev | AAAACTGCAGAGTGGCGCGATATTTTAACG |
| YNPR1-Term-Xba-for | CTAGTCTAGACAATAAATAAGCGTCCCCAC |
